# Advancing the measurement of favorable selection in Medicare Advantage: evidence from encounter data

**DOI:** 10.1093/haschl/qxag182

**Published:** 2026-07-10

**Authors:** Debra Bozzi, Ariel Caplan, Jared Doom, Emily Boudreau, Mike Hartjes, Richard Schwartz, Yong Li, Ashley Ray, Gosia Sylwestrzak

**Affiliations:** Humana Healthcare Research, Louisville, KY 40202, United States; Humana, Inc., Louisville, KY 40202, United States; Humana, Inc., Louisville, KY 40202, United States; Humana Healthcare Research, Louisville, KY 40202, United States; Humana, Inc., Louisville, KY 40202, United States; Humana, Inc., Louisville, KY 40202, United States; Humana Healthcare Research, Louisville, KY 40202, United States; Humana, Inc., Louisville, KY 40202, United States; Humana Healthcare Research, Louisville, KY 40202, United States

**Keywords:** Medicare, Medicare Advantage, favorable selection, payment policy

## Abstract

**Introduction:**

Debate persists over how favorable selection in Medicare Advantage (MA) affects federal spending relative to Traditional Medicare (TM). Prior studies, including the Medicare Payment Advisory Commission's (MedPAC's) March 2025 Report, focus on TM-to-MA switchers—roughly one-third of MA enrollees—but extrapolating these findings to the broader MA population is sensitive to identifying assumptions because switchers’ cost patterns may differ from non-switchers. This study uses MA encounter data to compare switchers and non-switchers and assess an alternative extrapolation approach to estimating favorable selection.

**Methods:**

We estimated favorable selection among 1.4 million MA enrollees from 2020 to 2022, generating separate estimates for switchers and non-switchers. Using a 5% national Medicare sample, we attempted to replicate MedPAC's switcher-based analysis, then modified its extrapolation by comparing risk-adjusted costs (imputed with TM claims) between switchers and non-switchers using MA encounter data.

**Results:**

Favorable selection ranged from 4.9% to 5.8% between 2020 and 2022—32% to 40% lower than our attempted replication of MedPAC's estimate (8.2%-8.8%).

**Conclusion:**

Using an alternative extrapolation approach lowered favorable selection, underscoring its sensitivity to methods and the importance of further refinement for MA payment policy.

Key points

- We updated the Medicare Payment Advisory Commission's (MedPAC's) March 2025 approach to estimating favorable selection in Medicare Advantage (MA) by directly comparing risk-adjusted imputed costs between individuals who switched from MA to Traditional Medicare (“switchers”) and all other MA enrollees (“non-switchers”).
- Revising MedPAC's extrapolation method, this study estimates that favorable selection increased MA payments by 4.9%-5.8% for payment years 2020-2022—32%-40% lower than our attempted replication of MedPAC's March 2025 estimate (8.2%-8.8%).
- Lower estimates were partly driven by differences in population mix; non-switchers (approximately two-thirds of the MA population) had higher risk-adjusted costs at younger ages than comparable switchers, reducing the magnitude of favorable selection.

## Introduction

There is growing dialogue among policymakers and healthcare stakeholders on the role of favorable selection in Medicare Advantage (MA). Favorable selection occurs when beneficiaries who choose to enroll in MA are systematically less costly than expected, even after accounting for their risk scores.^1,2^ This phenomenon has important implications for Medicare's solvency, as MA plans are paid a capitated, per-member per-month (PMPM) rate based on average spending in Traditional Medicare (TM). If MA enrollees cost less than their TM counterparts with the same risk score, these payments may exceed the actual cost of care, resulting in higher payments to MA plans and potentially inefficient allocation of government funds.^2^ With more than half of Medicare-eligible beneficiaries now enrolled in MA plans,^3^ it is increasingly critical to understand the extent of favorable selection in MA to inform policies on payment equity in Medicare.

Measuring favorable selection is complex, and estimates vary across studies and over time.^4-17^ While research from the early-to-mid 2000s suggested that reforms to the risk-adjustment system—such as implementation of the Centers for Medicare and Medicaid Services Hierarchical Condition Category (CMS-HCC) model—had largely addressed longstanding concerns about favorable selection,^8-11,15^ recent growth in MA enrollment and increasing scrutiny of MA payment practices have prompted new analyses examining favorable selection and its implications for Medicare spending. Newer estimates indicate that favorable selection increased MA payments by 10% in 2022, according to the widely referenced MedPAC March 2025 Report,^5^ with a University of Southern California Schaeffer (USC) Institute analysis suggesting an even higher impact of 14.4%.^7^

In principle, favorable selection could be assessed by comparing risk-adjusted spending between MA and TM enrollees, but several challenges limit this approach.^4^ Notably, we cannot observe what MA enrollees would have cost in TM. While TM claims provide detailed information on TM expenditures and utilization, no equivalent source exists for MA. MA encounter data—the closest alternative—lacks payment information and may be incomplete in capturing all services delivered to beneficiaries, with large variation across insurers.^18^ Although CMS initiatives have improved encounter data quality and transparency in recent years, important limitations persist, requiring rigorous validation before reliably using these data in research.^19-21^ Disentangling favorable selection from other MA–TM differences is also challenging. MA plans have stronger incentives to document diagnoses, potentially inflating risk scores.^5,6,22^ MA's care management practices may lead to more efficient treatment of patients, further complicating comparisons.^4,16,23^ Additional factors, such as geographic variation in MA plan offerings and provider networks,^24-26^ may also influence observed spending gaps.

To address these challenges, many studies (including those conducted by MedPAC) focus on beneficiaries who switch from TM to MA (“switchers”).^8,12,27^ By comparing the pre-switch, risk-adjusted costs of these individuals to those who remain in TM (“stayers”), researchers can isolate selection effects for this specific group.^5-7^ Because switchers encompass only one-third of the MA population, estimates must be extrapolated to the broader MA population, including MA beneficiaries who did not switch into MA from TM (“non-switchers”)^5^ to ensure that findings are relevant for policy discussions. MedPAC extrapolates favorable selection using external evidence to suggest similar selection patterns between switchers and non-switchers, adjusting for decedent status and enrollment duration in MA.^8,28,29^ These adjustments focus on a limited set of characteristics and may not fully reflect differences in health status, demographics, or behaviors that influence spending, and in turn, selection.^5^ Previous research shows demographic and clinical differences between switchers and non-switchers, suggesting the importance of considering the full scope of these differences in extrapolation.^30-32^

This is the first analysis to use MA encounter data to directly compare risk-adjusted spending (with costs imputed using TM claims) between switchers and non-switchers, presenting an alternative approach to estimating favorable selection. In this study, we attempted to replicate MedPAC's approach for estimating favorable selection among switchers to evaluate the impact of our methodology and most effectively contribute to policy discussions, then built on this foundation by modifying the extrapolation approach, directly measuring imputed risk-adjusted spending.

## Data and methods

### Conceptual framework for estimating favorable selection

Favorable selection reflects the extent to which MA enrollees’ expected TM spending falls below the level predicted by the CMS-HCC risk-adjustment model. To measure this, we follow a framework consistent with MedPAC, comparing pre-switch, risk-adjusted costs of MA switchers and TM stayers, attributing the difference to selection among switchers.^5-7^ We then extrapolate this switcher-based estimate to the remaining MA population by combining the results for switchers and non-switchers, yielding an overall estimate of favorable selection.

MedPAC's March 2025 Report estimates favorable selection among switchers by comparing their risk-adjusted spending in TM (in the year before the switch) with the spending level for stayers, adjusting for any attenuation in selection due to regression-to-the-mean. Risk-adjusted spending is calculated by dividing the PMPM costs for Medicare Parts A and B by the average CMS-HCC risk score within each population. We attempt to replicate MedPAC's approach for the switcher population to focus on extrapolation, where there was the greatest opportunity to test methodological sensitivity. Because MedPAC only reports an overall estimate of favorable selection (rather than separate estimates for switchers and non-switchers), we highlight our attempted replication of MedPAC's analysis alongside our result to facilitate direct comparison of switcher and non-switcher estimates.

To estimate favorable selection among non-switchers, MedPAC draws on external evidence to generalize findings from switchers to the broader MA population,^5,28,29^ adjusting for differences in MA enrollment duration and decedent status. Newhouse et al. used mortality as a proxy for favorable selection and found substantially lower mortality rates among enrollees who chose MA in their first year of Medicare eligibility vs those who chose TM.^28^ Teigland et al. examined pre-Medicare spending between 2015 and 2019, finding 12% lower risk-adjusted spending among MA enrollees relative to TM.^29^ Although these studies do not directly assess selection effects, their estimates of pre-Medicare spending and risk scores are used to infer selection by risk-standardizing spending differences and translating them into payments above TM spending. We focus on MedPAC's 2025 methodology; however, the March 2026 report^33^ also accounts for pre-Medicare spending differences among dual-eligibles, from Pelech et al.^17^

Our extrapolation method differs from MedPAC's by using MA encounter data to directly measure differences in risk-adjusted costs (imputed using TM claims) between switchers and non-switchers. Because MedPAC lacked direct information on non-switchers’ costs and risk scores, it relied on stratification over 2 characteristics—enrollment duration and mortality status—to attempt to capture unobserved population-level differences. In contrast, our method directly estimated imputed risk-adjusted spending, eliminating the need for such stratification. To identify where the risk adjustment model may incompletely capture beneficiary cost differences at the point of MA enrollment, we avoid adjusting for factors endogenous to the model—whether explicitly embedded (eg, morbidity, age, sex, disability) or implicitly captured (eg, decedent status). Instead, we adjust for factors not captured by the risk model, including MA enrollment effects (eg, utilization management, provider networks) via plan mix and geographic variation.

Each extrapolation approach has tradeoffs. Our approach uses data that directly reflects observed differences in risk-standardized spending to estimate relative selection between switchers and non-switchers during the year of MA enrollment, which is a key strength. However, these estimates may also capture confounding from factors that are difficult to fully adjust for, including differences in utilization management, encounter data completeness, and coding intensity. In contrast, MedPAC's 2025 method is not affected by coding intensity and other plan-level features, and instead relies on post-enrollment selection among switchers to extrapolate to non-switchers (approximately two-thirds of MA enrollees), an approach that depends on external evidence of favorable selection among non-switchers^28,29^ and remains a key limitation.

### Data and study population

In our attempt to replicate MedPAC's estimate of favorable selection for TM-to-MA switchers, we used the Medicare Limited Data Set Standard (LDS) Analytical Files from a 5% random sample of Medicare beneficiaries from January 1, 2008 to December 31, 2022. We identified annual cohorts of beneficiaries who switched from TM to MA between January 1, 2010 and December 31, 2022. Beneficiaries were required to have at least 2 full years of continuous enrollment in TM Parts A and B prior to MA enrollment. Eligible beneficiaries were assigned to a reference year, defined as the year before switching to MA. We compared this population of switchers to stayers who remained in TM in the same reference year. Beneficiaries were excluded from the study on a monthly basis if they did not have Parts A and B coverage, were enrolled in hospice, were eligible for Medicare due to end-stage renal disease, had Medicare as a secondary payer, or lived outside of the 50 United States or Washington, DC.

To extrapolate findings from switchers to the broader MA population, we used MA encounter data from January 1, 2020 to December 31, 2022 for all MA beneficiaries, including those identified as switchers (described above) as well as those who were not identified as switchers (“non-switchers”) between January 1, 2010 and December 31, 2022. We used LDS claims from January 1, 2009 through December 31, 2021 to measure pre-switch cost patterns. Because switchers were identified separately in the LDS and MA encounter data, the samples may not be perfectly identical; however, to the best of our knowledge, they largely capture the same beneficiaries, as evidenced by stable Health Insurance Claim (HIC) number-based sampling and consistent demographic characteristics. Non-switchers included individuals who had never been enrolled in TM and individuals who were previously enrolled in TM but did not have sufficient enrollment history prior to switching. We excluded newly eligible MA enrollees, defined as those with demographic-only risk scores, to improve comparability between MedPAC's TM stayer population and MA non-switchers (details available in the Supplementary material, section 1).

### Imputing the TM-equivalent cost to MA encounter data

To address the lack of spending information in the MA encounter data, we adapted the Jung et al.^34^ method of applying standardized TM prices to services observed in MA encounters. Using TM claims, we applied the TM allowed amounts by service category (Inpatient, Outpatient, Carrier, Durable Medical Equipment, Skilled Nursing Facility [SNF], and Home Health) to the MA encounter data. We aggregated these costs at the county level and adjusted for service mix and developed logic for SNF and Home Health claims to price MA encounters using the TM payment system framework.

To assess the accuracy of our imputation approach, we compared imputed allowed costs vs actual allowed costs on the TM population at the member level, finding that nearly 70% (69.4%) of imputed claims were within 5% of the actual allowed amount. On average, imputed and actual allowed costs differed by 0.2%, with over- and under-predictions distributed evenly (Supplementary material, section 2).

### Estimating favorable selection among TM-to-MA switchers

For payment years 2020 through 2022, we estimated favorable selection for TM-to-MA switchers by following, to the extent possible, the methodology described in MedPAC's March 2025 Report.^5^ We compared risk-adjusted monthly spending between MA switchers and TM stayers in the same reference year, with separate estimates for each cohort of switchers. We then measured regression-to-the-mean within each cohort to capture the extent to which selection percentages converged toward average levels over time. We calculated the selection percentage by examining the ratio of average monthly risk-adjusted spending between MA switchers and TM stayers. Values below 100% indicate favorable selection into MA, and values above 100% indicate unfavorable selection into MA. We then calculated the impact on MA payments by taking the reciprocal of the selection percentage and subtracting by 1.

Although our goal was to replicate MedPAC's approach, our estimates may differ due to data constraints and gaps in the explanation of methods in MedPAC's report. Most notably, we were limited to using the 5% LDS sample starting in 2008, while MedPAC used the 100% Innovator Data Set beginning in 2006. The smaller sample reduced reliability among older beneficiaries (as sample sizes declined) and constrained replication of certain steps, particularly when estimating initial selection. A detailed comparison of our approach vs MedPAC's can be found in the Supplementary material, section 3.

### Extrapolating favorable selection to the full MA population

We extrapolated results from switchers to non-switchers, implementing 2 methods: (1) an attempted replication of MedPAC's 2025 method, and (2) an alternative approach that directly compared risk-adjusted imputed spending between the 2 groups using MA encounter data.

Our attempted replication of MedPAC's 2025 approach stratified switchers and non-switchers by year of MA entry and decedent status, including those who died in the payment year (decedents), those who died in the year after the payment year (near-decedents), and those who did not die within 2 years of the payment year (non-decedents). Within each stratum, we assumed the same selection percentage for switchers and non-switchers (generalized from the switcher-based estimate and MedPAC's application of external studies showing lower pre-Medicare spending for newly eligible MA vs TM enrollees^8,29^). Selection percentages for the non-switchers were aggregated by taking the weighted sum across all subgroups, with weights calculated by multiplying member months by the average CMS-HCC risk score.

In contrast, our approach used encounter data to directly estimate risk-adjusted imputed spending for both switchers and non-switchers. Imputed spending was calculated using the methods described earlier, and risk-adjusted costs were derived by dividing average imputed spending from both populations by the average CMS-HCC risk score. Risk scores for both populations were computed from MA encounter data and TM claims diagnoses (where applicable), as well as the Master Beneficiary Summary File. By measuring both risk scores and imputed spending directly—rather than extrapolating selection patterns based on enrollment duration or mortality status—this approach yields a population-wide estimate of favorable selection that naturally reflects observed differences between switchers and non-switchers.

To isolate favorable selection from post-enrollment plan effects (eg, utilization management, benefit design, provider networks), we applied 2 adjustments to risk-adjusted spending. First, to account for plan characteristics that influence spending after enrollment, we standardized spending for differences in plan mix (ie, the share of beneficiaries enrolled in Health Maintenance Organization [HMO], Preferred Provider Organization [PPO], and other product lines) by reweighting non-switchers to match the switcher distribution. Second, consistent with our attempted replication of MedPAC's switcher analysis, we standardized county-level costs using the 2020 through 2022 CMS Ratebook area adjustment factors. While favorable selection cannot be fully separated from plan-driven influences on utilization, risk scores, and risk-standardized spending, these adjustments are designed to reasonably address these factors.

Coding intensity is another important factor that contributes to post-enrollment plan effects. In this framework, uniform coding adjustments across groups do not affect results; only relative differences between switchers and non-switchers matter. Given ongoing debate in recent literature^35,36^ on coding intensity estimates, we applied no coding adjustment to MA risk scores in our main analysis and instead tested sensitivity to differential coding between switchers and non-switchers (sensitivity analyses described later).

After applying these adjustments, we estimated favorable selection for the broader MA population. We first derived the favorable selection rate for non-switchers. To do this, we calculated the implied TM-level cost—that is, the inferred average TM risk-adjusted cost—by dividing the switchers’ imputed, risk-adjusted monthly spending by their selection percentage (obtained from our attempted replication of MedPAC's analysis for switchers). We then divided non-switchers’ imputed, risk-adjusted monthly spending by the implied TM-level cost to calculate their selection percentage. We estimated the impact of favorable selection on MA payments separately for switchers and non-switchers by taking the reciprocal of each group's selection percentage and subtracting by 1. Total MA favorable selection was then calculated by taking an enrollment-weighted average of imputed costs and risk scores across the 2 groups to determine the selection percentage and favorable selection estimate (as described above). A comparison of MedPAC's approach vs this study's is shown in Table 1.

**Table 1:** Comparison of MedPAC's March 2025 methodology vs this study's approach to estimating favorable selection.

Phase of favorable selection calculation	Analysis component	MedPAC (March 2025)	This study
**MA-to-TM switchers analysis**	Data set	100% TM data from the innovator data set	5% TM sample from the limited data set
	Study period	2006-2022	2008-2022
	Study sample	TM-to-MA switchers from 2008 to 2022	TM-to-MA switchers from 2010 to 2022
	Initial selection	Compares pre-switch costs between TM-to-MA switchers, compared to those remaining in TM	Same as MedPAC's March 2025 Report
	Regression-to-the-mean	Assumes switchers’ selection percentages approach toward mean at same rate as proxy cohort of those remaining in TM	Same as MedPAC's March 2025 Report
	Accounting for geographic variation	Matched MA switchers with TM stayers at the county-level. Aggregate results using switchers’ county enrollment as weight	Used the 2020-2022 CMS Ratebook, which measures county-level differences in risk adjusted TM costs
**Extrapolation**	Data source	100% TM innovator data	MA encounter data with TM-based cost imputation
	Study population	MA non-switchers, 2020-2022	MA non-switchers, 2020-2022 (excluding new-to-Medicare^a^)
	Basis for non-switcher selection estimates	Extrapolates from switchers using parameters from external studies^b^	Direct comparison of risk-adjusted, imputed costs between switchers and non-switchers
	Stratifies separately across subpopulations	Stratifies by decedent status and MA enrollment year^c^	Does not stratify on factors endogenous to the risk model
	Geographic adjustment	Aggregates MA-TM comparisons over switcher county distribution	Standardizes costs using 2020-2022 CMS Ratebook county factors
	Plan mix adjustment	No adjustment	Adjusts for HMO/PPO/other plan mix.
	Coding intensity adjustment	None	None; tested varying levels of relative coding between switchers and non-switchers through sensitivity analyses
**Overall favorable selection**	Calculation of selection percentages	Calculated as the ratio of average monthly risk-adjusted spending between all MA enrollees and TM stayers	Same as MedPAC's March 2025 Report
	Impact of favorable selection on MA payment	Calculated as the reciprocal of the selection, percentage and subtracting by 1	Same as MedPAC's March 2025 Report

MedPAC's March 2026 Report also cites the following study showing pre-Medicare spending differences by dual-eligibility: Pelech et al.^17^

^a^New-to-Medicare enrollees are defined as those with demographic-only risk scores.

^b^MedPAC's March 2025 Report, the main comparison to this study, cites the following external literature showing lower pre-Medicare spending among those who select MA vs TM at eligibility: Teigland et al.^29^ and Newhouse et al.^28^

^c^MedPAC's March 2026 Report also extrapolates separately by dual-eligibility status.

The adjustments described above implicitly assume that post-enrollment plan effects impact switchers and non-switchers equally. To assess this assumption, we conducted several sensitivity analyses. First, we assessed whether our plan mix adjustment adequately captured differences in “MA exposure”—that is, the effects of MA enrollment (eg, utilization management, benefit design, provider networks), defined for switchers as the ratio of encounter-based to TM claims-based PMPM costs and risk scores (based on MedPAC's methodology for estimating TM counterfactuals), calculated separately. This approach relies on the premise that risk-adjusted spending for MA switchers can be estimated using both TM claims and MA encounter data. A comparison of MA encounters vs TM claims in this study sample yields a 7.9% increase in coding intensity (net of CMS's 5.9% coding adjustment) and a 5.1% reduction in spending among switchers in MA encounter data vs TM claims. Overall, these estimates are directionally consistent with prior literature,^5,29,33,37,38^ though they depend on modeled TM counterfactuals and may diverge from actual exposure levels. The coding effect appears plausible,^5,33,37^ while the spending effect reflects the combined influence of utilization management, prior authorization, network design, and differences between encounter data and TM claims, which cannot be disentangled.^29,33,38^ Our main analysis applies the same exposure effect to both switchers and non-switchers, while sensitivity analyses vary the assumed exposure for switchers vs non-switchers to assess impacts on favorable selection.

Second, we examined results without plan mix and geographic standardization to show how these adjustments affect our overall estimate of favorable selection. Third, we tested how differential coding practices altered estimated selection across several scenarios: (1) higher uniform coding intensity (ie, to demonstrate that only relative coding differences between switchers and non-switchers impact estimated selection); (2) no coding intensity among first-year MA enrollees (These analyses applied a series of overall coding adjustments: (1) the 5.9% overall adjustment used by CMS in actual payment calculations, (2) 10.0%, and (3) 15.0%.); (3) higher coding intensity for dual-eligibles (This analysis applied 12.7% to long-term institutionalized enrollees (regardless of dual status), 15.5% for non-duals, 30.1% for partial duals, 20.8% for full duals, aligned with MedPAC's July 2025 Data Book.)^39^; and (4) coding intensity that increased with MA enrollment duration.^40^

Because new-to-Medicare beneficiaries are excluded, the “no-first-year coding” scenario applies only to first-year switchers, effectively lowering their coding intensity relative to non-switchers. We retain this as an illustrative sensitivity analysis, though it may not reflect coding patterns in the broader MA population. Similarly, the enrollment duration analysis is based on evidence from switchers and may not generalize to non-switchers. Estimating appropriate coding effects would require distinct growth trajectories across these groups, for which no empirically validated methods exist. We therefore present this as an illustrative sensitivity check, recognizing that it may not reflect true coding changes over time. Finally, in the dual-eligible coding intensity scenario, switchers exhibit higher coding intensity than non-switchers due to their higher share of duals (Supplementary material, section 4).

### Exploratory post hoc analysis

To understand why our favorable selection estimate differs from MedPAC's, we compared population characteristics of switchers and non-switchers, focusing on age, decedent status, and the associated risk-adjusted spending for each group. Because MedPAC assumed equal selection for switchers and non-switchers within the same decedent group and MA entry year, any population differences that affect spending patterns and remain unaccounted for can substantially impact favorable selection estimates.

We then tested the impact of certain demographic factors on favorable selection estimates. To do this, we replicated MedPAC's extrapolation approach with one key difference—in addition to matching on MA entry year and decedent status, we also controlled for age. We also compared this estimate to MedPAC's 2026 published favorable selection estimate, which controlled for dual status, in addition to MA entry year and decedent status.

## Results

### MA population characteristics

For the payment year 2022, among 1 402 508 MA beneficiaries, 33.9% were TM-to-MA switchers, while the remaining 66.1% comprised non-switchers. Non-switchers tended to be younger, with 62.4% of the population under age 75, compared with 47.5% of switchers, and were less likely to qualify for Medicare due to disability (20.5% vs 35.7%). Table 2 shows beneficiary characteristics for the full MA sample in 2022.

**Table 2:** MA population characteristics; a comparison of switchers vs non-switchers 2022.^a^

	Overall MA population	Switchers	Non-switchers
# of Beneficiaries	1 402 508	475 813	926 725
# of beneficiary months	*n* = 16 163 041	*n* = 5 340 368	*n* = 10 822 673
Female; *n*(%)	9 114 291 (56.4%)	2 965 794 (55.5%)	6 148 497 (56.8%)
Age; *n*(%)			
<65	2 033 060 (12.6%)	937 071 (17.5%)	1 095 989 (10.1%)
65-74	7 262 616 (44.9%)	1 601 347 (30.0%)	5 661 269 (52.3%)
75-84	5 158 245 (31.9%)	2 062 556 (38.6%)	3 095 689 (28.6%)
85+	1 709 120 (10.6%)	739 394 (13.8%)	969 726 (9.0%)
Race; *n*(%)			
Black	2 180 007 (13.5%)	846 869 (15.9%)	1 333 138 (12.3%)
White	12 055 686 (74.6%)	4 005 198 (75.0%)	8 050 488 (74.4%)
Underrepresented	1 927 348 (11.9%)	488 301 (9.1%)	1 439 047 (13.3%)
Dual-eligible; *n*(%)	3 818 454 (23.6%)	1 709 224 (32.0%)	2 109 230 (19.5%)
Original reason for Medicare; *n*(%)			
Age	12 023 082 (74.39%)	3 428 425 (64.2%)	8 594 657 (79.4%)
Disability	4 124 566 (25.52%)	1 906 690 (35.7%)	2 217 876 (20.5%)
ESRD	6943 (0.04%)	1505 (0.0%)	5438 (0.1%)
Disability and ESRD	8450 (0.05%)	3748 (0.1%)	4702 (0.0%)
Plan type			
HMO	9 533 122 (59.0%)	2 630 281 (49.3%)	6 902 841 (63.8%)
PPO	6 427 271 (39.8%)	2 636 840 (49.4%)	3 790 431 (35.0%)
Other	202 648 (1.3%)	73 247 (1.4%)	129 401 (1.2%)
Rural; *n*(%)	2 370 483 (14.7%)	1 070 222 (20.0%)	1 300 261 (12.0%)
CMS-HCC risk score^b^; mean [SD]	1.238 [1.106]	1.332 [1.157]	1.192 [1.078]
Decedent status; *n*(%)			
Decedent	315 261 (2.0%)	129 928 (2.4%)	185 333 (1.7%)
Near-decedent	618 238 (3.8%)	255 495 (4.8%)	362 743 (3.4%)
Non-decedent	15 229 542 (94.2%)	4 954 945 (92.8%)	10 274 597 (94.9%)

^a^Population characteristics for 2020 and 2021 cohorts can be found in the Supplementary material.

^b^All MA risk scores expressed prior to CMS 5.9% coding intensity factor adjustment.

### Favorable selection estimate

**Figure 1:**
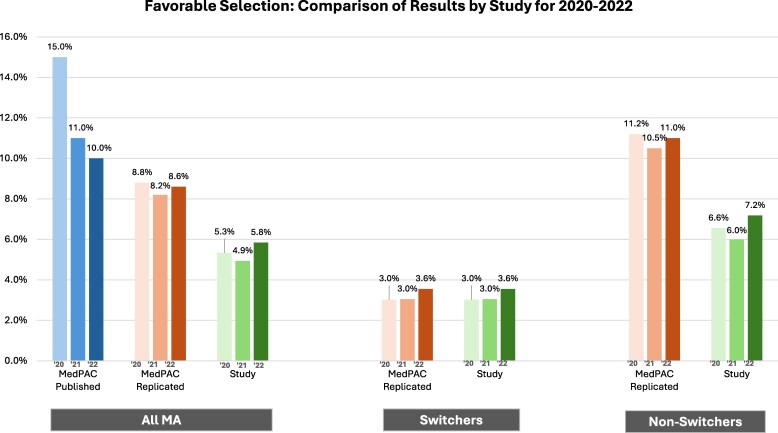
Overall impact of favorable selection on MA payments: the present study vs MedPAC's 2025 method, 2020-2022.

From 2020 to 2022, we estimate that favorable selection increased MA payments by 4.9%-5.8%, compared with MedPAC's published range of 10.0%-15.0% in its March 2025 Report. Our replication of MedPAC's analysis produced lower estimates of 8.2%-8.8%. Thus, our approach represented a 32%-40% annual decrease in estimated favorable selection relative to our attempted replication of MedPAC's analysis (Figure 1).

For the switcher population, our attempted replication of MedPAC's analysis yielded favorable selection estimates that ranged from 3.0% to 3.6% between 2020 and 2022. In extrapolating to non-switchers, we observed an increase in overall favorable selection of 1.9-2.3 percentage points (pp) annually. In comparison, our attempted replication of MedPAC's extrapolation found a greater increase in favorable selection, by 5.1-5.8 pp per year. In both analyses, extrapolation increased total estimated favorable selection among the MA population, but the impact is differentially lower in the present study (Figure 1). (Our attempt to replicate MedPAC's favorable selection estimate switchers yielded a result that differed from the actual published estimates in the March 2025 Report. Discrepancies are due to the methodological differences outlined in the material.)

We found that non-switchers had lower risk-adjusted, imputed PMPM costs ($639-770), than switchers ($661-792) and TM beneficiaries (calculated as the implied TM-level cost) ($680-816). These lower costs resulted in higher estimated favorable selection among non-switchers (6.0%-7.2%) than among switchers (3.0%-3.6%). This pattern aligned directionally with MedPAC's finding, which also showed greater favorable selection among non-switchers. After applying enrollment weights, extrapolating from switchers to the broader MA population raised the overall favorable selection estimate, but only modestly (1.9%-2.3%) (Table 3).

**Table 3:** Detailed calculation of favorable selection estimate in MA (2020-2022).

Impact of favorable selection extrapolation by year					
		2020	2021	2022	
*(1)*	Switchers’ selection percentage	97.1%	97.0%	96.6%	
**MA encounter data—switchers**					
*(2)*	Member months	4 226 627	4 831 606	5 340 368	
*(3)*	Net PMPM @ TM cost-sharing levels	$874.09	$995.41	$989.29	
*(4)*	Geographic adjustment	0.988	0.984	0.984	
*(5)*	Geo-adjusted net PMPM	$884.79	$1011.32	$1005.37	*= (3)/(4)*
*(6)*	Average risk score^*^	1.340	1.278	1.332	
*(7)*	Risk-adjusted net PMPM	$660.50	$791.59	$754.95	*= (5)/(6)*
*(8)*	Implied TM costs (after MA exposure to costs)^#^	**$680.46**	**$815.71**	**$781.78**	*= (7)/(1)*
**MA encounter data—non-switchers**					
*(9)*	Member months	9 392 852	10 065 009	10 822 673	
*(10)*	Net PMPM @ TM cost-sharing levels	$727.24	$842.04	$819.55	
*(11)*	Geographic adjustment	0.970	0.972	0.972	
*(12)*	Geo-adjusted net PMPM	$749.63	$866.07	$842.99	*= (10)/(11)*
*(13)*	Average risk score	1.209	1.154	1.192	
*(14)*	Plan-mix-adjusted net PMPM	$751.24	$864.49	$846.91	
*(15)*	Plan-mix-adjusted risk score^*^	1.176	1.123	1.161	
*(16)*	Risk-adjusted net PMPM	$638.59	$769.58	$729.43	*= (14)/(15)*
*(17)*	Non-switchers’ selection percentage	93.8%	94.3%	93.3%	*= (16)/(8)*
**MA encounter data—total population**					
*(18)*	Member months	13 619 479	14 896 615	16 163 041	*= (2)* *+* *(9)*
*(19)*	Adj. net PMPM @ TM cost-sharing levels	$792.69	$912.11	$899.27	*= {(2) × (5) + (9) × (14)}/(18)*
*(20)*	Average risk score^*^	1.227	1.173	1.217	*= {(2) × (6) + (9) × (15)}/(18)*
*(21)*	Risk-adjusted net PMPM	$646.01	$777.35	$738.65	*= (19)/(20)*
*(22)*	Total MA population selection percentage	94.9%	95.3%	94.5%	*= (21)/(8)*
**Impact of favorable selection on MA payments (%)**					
*(23)*	Switchers	3.0%	3.0%	3.6%	*= 1/(1) − 1*
*(24)*	Non-switchers	6.6%	6.0%	7.2%	*= 1/(17) − 1*
*(25)*	**All MA**	**5.3%**	**4.9%**	**5.8%**	*= 1/(22) − 1*

^*^All MA risk scores expressed prior to CMS 5.9% coding intensity factor adjustment.

^#^The implied TM cost is defined as the inferred average TM risk-adjusted cost (after MA exposure to costs).

Our estimates showed sensitivity to alternative assumptions about post-enrollment plan effects for switchers vs non-switchers; however, results remained below MedPAC's published 2025 estimate across all scenarios. A 1 pp increase in MA claims exposure and a 2 pp increase in risk score exposure for switchers (relative to non-switchers) reduced the estimate by 0.6 pp. Conversely, a 1 pp increase in risk score exposure and a 2 pp increase in claims exposure raised the estimate by 1.3 pp, underscoring the sensitivity of our results to these assumptions.

Removing plan mix and geographic standardization had a larger effect, increasing estimates by 2.6-3.4 pp and underscoring sensitivity of results to methodological choices. These adjustments are retained as core elements of the analysis because they address factors outside the risk model that may affect risk-adjusted spending independently of selection. Favorable selection estimates were unchanged using any uniform coding adjustment, but declined modestly (0.6-1.0 pp) with no coding adjustment for first-year TM-to-MA switchers, and by 2.6 pp when coding intensity increased with MA tenure. Estimates increased by 0.8 pp under assumptions of higher coding intensity for dual-eligibles (Supplementary material, section 4).

### Exploratory post hoc analysis

**Figure 2:**
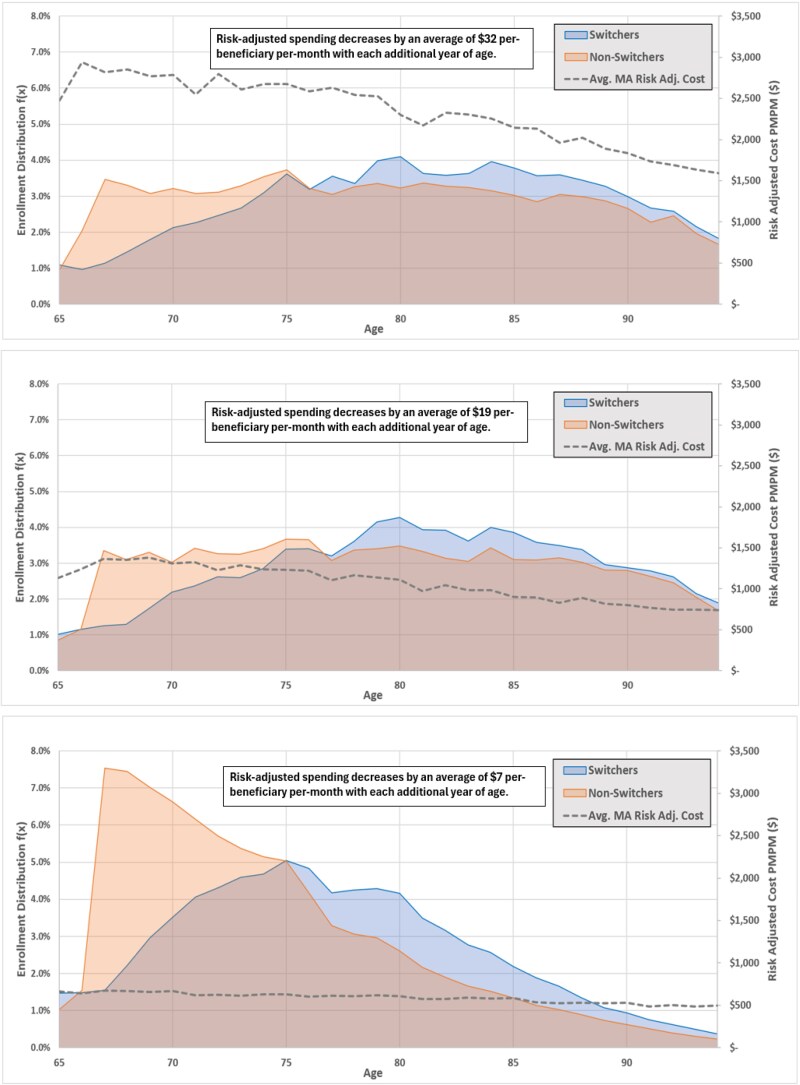
Average MA risk-adjusted spending and member month distribution of switchers vs non-switchers by age and decedent status, 2020–2022^*#^. *Panel A. Decedents. Panel B. Near-decedents*. *Panel C. Non-decedents*. *The sample is restricted to beneficiaries aged 65+ to capture the majority of enrollment and avoid low credibility per-beneficiary per-month cost estimates. ^#^All MA risk scores are applied prior to CMS 5.9% coding intensity factor adjustment.

In exploratory post hoc analyses, we examined population differences between switchers and non-switchers to understand possible drivers of our lower favorable selection estimate relative to MedPAC's. We found that across all decedent groups, a higher share of individuals in the non-switcher population were under age 75 compared with those in the switcher population (Figure 2). Further, there was an inverse relationship between age and average risk-adjusted spending among MA enrollees, where younger beneficiaries had higher risk-adjusted costs compared with older beneficiaries. The difference in average risk-adjusted spending between younger and older MA enrollees was most pronounced within decedents (Figure 2, Panel A), yet this pattern persisted across all decedent groups (Figure 2, Panels B and C). Among decedents, beneficiaries under age 75 had an average risk-adjusted spending level of $2658 compared with beneficiaries age 75 or older, who had an average risk-adjusted spending level of $2148. These differences in risk-adjusted spending may be partially reflective of differences in utilization management and/or coding intensity, rather than favorable selection alone.

The larger share of younger beneficiaries among non-switchers—particularly within the decedent and near-decedent subgroups—and their higher risk-adjusted spending have important implications for estimating favorable selection. To quantify the magnitude of this effect—using a methodology that is unaffected by utilization management and coding intensity—we attempted to replicate MedPAC's extrapolation approach with an added control for age-related spending differences. Incorporating this adjustment reduced estimated favorable selection by 3.0 pp relative to our attempted replication of MedPAC's 2022 result (5.6% vs 8.6%, respectively). These findings demonstrate the importance of accounting for granular differences in risk-adjusted spending across subpopulations, especially by age, and underscore the sensitivity of favorable selection estimates to assumptions about population composition. In contrast, controlling instead for dual-eligibility status, consistent with MedPAC's 2026 published estimate,^33^ did not materially change the estimate (11.0%), suggesting that age is a more influential driver of favorable selection differences (Supplementary material, section 5).

## Discussion

In the first analysis to use MA encounter data to estimate favorable selection, we find that favorable selection increased MA payments by 4.9%-5.8% between 2020 and 2022—32%-40% lower than our attempted replication of MedPAC's March 2025 estimate (8.2%-8.8%) and 42%-65% lower than MedPAC's published March 2025 estimate (10.0%-15.0%). These differences largely reflect our use of encounter data to directly assess imputed, risk-adjusted spending across the broader MA population: extrapolation increased estimated favorable selection by only 1.9-2.3 pp per year, compared with 5.1-5.8 pp in our attempted replication of MedPAC's method. As a result, our analysis offers an alternative framework for comparing favorable selection between MA switchers and non-switchers and yields estimates that are notably lower than prior benchmarks ranging from 10%^5^ to 14.4%.^7^

The primary distinction across approaches lies in how selection is inferred. MedPAC extrapolates using parameters informed by external studies^28,29^ that examine pre-Medicare spending differences between beneficiaries who chose MA vs TM at Medicare eligibility; these differences, combined with risk scores, are used to estimate selection effects.^5^ In contrast, our analysis uses encounter data to directly compare risk-adjusted, imputed spending between switchers and non-switchers to estimate favorable selection across the broader MA population. This analysis makes a substantial contribution to the methodological discussion informing appropriate payment policy. MedPAC's reliance on the same external studies in its March 2024^6^ and 2025^5^ reports—despite its use of a different extrapolation approach—highlights the subjectivity inherent in translating external evidence into population-wide selection estimates. MedPAC has acknowledged this limitation, citing “uncertainty regarding the selection effect for MA enrollees outside of the [switchers] population…due to a lack of data.”^5^ Subsequent refinements in MedPAC's March 2026 Report,^33^ including adjustments for pre-Medicare spending among dual-eligible beneficiaries, further underscore the evolving nature of these methods and the continued uncertainty surrounding the magnitude of favorable selection.

While our method focuses on within-MA differences, it remains grounded in MedPAC's framework through comparisons between MA switchers and TM stayers, making MA-TM differences central to estimating selection. To improve comparability between our MA and TM populations, we exclude new-to-Medicare enrollees from our extrapolation, as these individuals are not included in MedPAC's TM stayer cohort. In contrast, because newly eligible beneficiaries are fully included in MedPAC's non-switcher population, their analysis relies on studies of these beneficiaries to infer selection among non-switchers more broadly. Consistent with the external literature cited by MedPAC,^28,29^ our findings indicate greater favorable selection among non-switchers than switchers (and by extension, the TM comparison population). However, the magnitude of favorable selection in our analysis is smaller than implied by MedPAC's extrapolation, which assumes that pre-Medicare spending differences carry forward into the post-Medicare setting; to the extent that benefit design and measurement of beneficiary risk differ, extrapolation based on pre-Medicare spending may miscalculate estimates of favorable selection. Differences in regression-to-the-mean between switchers and non-switchers may also affect the estimate. Moreover, while MedPAC stratifies its extrapolation by decedent status, the external studies do not directly evaluate whether selection effects vary by mortality status, providing limited empirical guidance for this dimension.

Post hoc analyses indicate that population mix plays an important role in estimating favorable selection. Non-switchers—especially decedents and near-decedents—have higher risk-adjusted spending than switchers, which contributes to lower estimated favorable selection. Even within MedPAC's extrapolation framework, adding an adjustment for age-related spending differences (beyond decedent status and MA enrollment duration) reduced favorable selection by 3.0 pp, highlighting the relevance of more detailed population controls. In contrast, accounting for dual-eligibility status in MedPAC's 2026 approach did not materially change its estimate, suggesting that age is more influential in estimating selection. These findings highlight the sensitivity of favorable selection estimates to data sources and extrapolation methods, especially across different demographic groups.

This study has several limitations. Most notably, reliance on encounter data limits our ability to fully disentangle favorable selection from plan-driven factors affecting observed utilization, risk scores, and risk-standardized spending. Although we adjust for MA exposure (via plan mix), and geography, residual differences between switchers and non-switchers may remain due to variation in encounter data completeness or accuracy, coding practices, imputation methodology, or plan-level features (eg, utilization management, network design, claims recovery). These data limitations are primarily concerning if they differentially affect switchers and non-switchers, which we cannot directly test. Our main analysis implicitly assumes equal MA exposure across groups, which may be reasonable if plan incentives are similar across populations. However, if switchers retain TM utilization patterns in MA, then exposure effects on coding and spending could be larger for non-switchers. We explore a range of scenarios with differential MA exposure between switchers and non-switchers, but these may not fully capture actual differences in enrollee experiences. Supplemental analyses for coding intensity and MA exposure, along with goodness-of-fit tests for cost imputation, suggest reasonable robustness; however, unmeasured plan-driven differences may remain. A notable exception was when we removed plan mix and geographic standardization, which increased estimated effects by 2.6-3.4 pp. Because these adjustments are intended to account for factors not captured by the risk model that may affect risk-adjusted spending independently of selection, they are retained as core components of the analytic framework. Consistent with prior studies,^41,42^ we address these concerns by interpreting results using encounter data cautiously and conducting extensive sensitivity analyses. Further, because any imputation approach could be affected by the limitations of encounter data, we anchored our estimates to Jung et al.'s^34^ validated, peer-reviewed methodology to minimize methodological bias.

Additionally, we were unable to fully replicate MedPAC's methodology for estimating favorable selection among switchers due to differences in data access and limited methodological detail in MedPAC's report. Our analysis used the 5% Limited Data Set starting in 2008, while MedPAC used the 100% Innovator Data Set beginning in 2006. The smaller sample also reduced reliability among older beneficiaries (as sample sizes declined). In addition, switchers were identified separately across the LDS and MA encounter data, so the samples may not contain identical beneficiaries; although the encounter data included roughly 2.5% fewer individuals, the demographic characteristics of the samples remained largely comparable. Finally, our analysis does not represent the full MA population, as we excluded new-to-Medicare enrollees to improve comparability between MA non-switchers and TM stayers. However, our analysis includes 90.1% of MA member-months, so results would likely generalize to the small remainder of the MA population.

Still, this study advances the literature by demonstrating the feasibility of using MA encounter data to estimate favorable selection in MA by directly measuring costs and risk scores for switchers and non-switchers. Our findings illustrate the tradeoffs inherent in different estimation approaches, underscoring the importance of continued methodological refinement as policymakers consider adjustments to MA payment policy.

## Supplementary Material

qxag182_Supplementary_Data

## Data Availability

The authors do not have permission to share the data.
