# Supplementary material for: Advancing the measurement of favorable selection in Medicare Advantage: evidence from encounter data
Source: Health Aff Sch. 2026 Jul 10;4(7):qxag182. doi: 10.1093/haschl/qxag182 (PMC13402953; doi:10.1093/haschl/qxag182)
Supplement: qxag182_Supplementary_Data [file qxag182_supplementary_data.zip › favorable_selection_ha_scholar_appendix_final.docx]

**Supplementary Material: Advancing the Measurement of Favorable Selection in Medicare Advantage: Evidence From Encounter Data**

Section 1: Study Population

Section 2: Cost Imputation

Section 3: Methodology for Estimating Favorable Selection Among TM-to-MA Switchers

Section 4: Sensitivity Analyses

Section 5: Exploratory Post-Hoc Analyses

**Section 1: Study Population**

***Beneficiary Waterfall Diagram (2022)*** ^§^

**
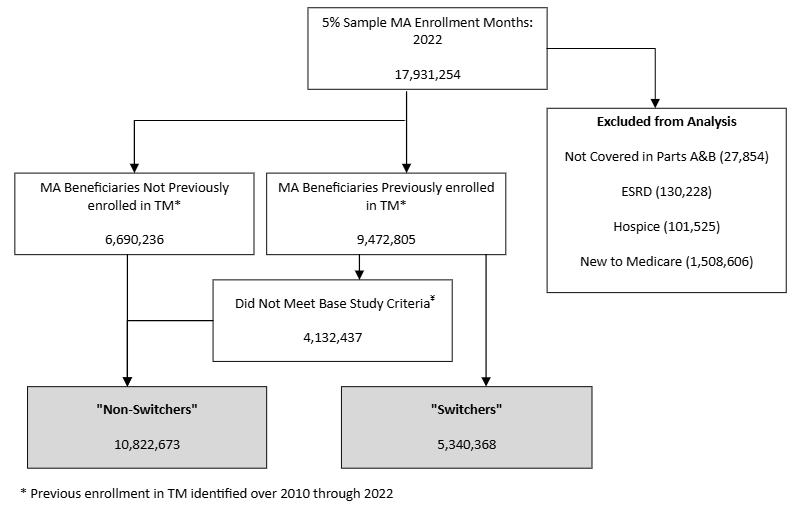
**

^*^ Previous enrollment in Traditional Medicare identified from 2010-2022

^§^ Beneficiaries are included only for months in which they are enrolled. For example, a non‑ESRD beneficiary eligible for Parts A and B all year but in hospice only in December 2022 is counted for 11 months and excluded for 1.

^¥^ Members did not meet base criteria for switchers if, for any month in the two calendar years prior to switching to MA, they had hospice status, ESRD status, Medicare as a secondary payer, were not eligible & enrolled in Medicare Part A, were not eligible & enrolled in Medicare Part B, or did not reside in a U.S. state or Washington, D.C.

***MA Population Characteristics: A Comparison of Switchers vs. Non-Switchers 2020, 2021***

**MA Population Characteristics, 2021**

|  | Overall MA Population | Switchers | Non-Switchers |
| --- | --- | --- | --- |
| # of Beneficiaries | 1,295,860 | 432,869 | 862,991 |
| # of Beneficiary Months | N = 14,896,615 | n = 4,831,606 | n = 10,065,009 |
| Female; n(%) | 8,417,587 (56.5%) | 2,688,627 (55.6%) | 5,728,960 (56.9%) |
| Age; n(%) |  |  |  |
| <65 | 1,920,108 (12.9%) | 858,914 (17.8%) | 1,061,194 (10.5%) |
| 65-74 | 6,707,255 (45.0%) | 1,475,988 (30.5%) | 5,231,267 (52.0%) |
| 75-84 | 4,656,778 (31.3%) | 1,834,352 (38.0%) | 2,822,426 (28.0%) |
| 85+ | 1,612,474 (10.8%) | 662,352 (13.7%) | 950,122 (9.4%) |
| Race; n(%) |  |  |  |
| Black | 1,985,725 (13.3%) | 765,400 (15.8%) | 1,220,325 (12.1%) |
| White | 11,188,124 (75.1%) | 3,635,664 (75.2%) | 7,552,460 (75.0%) |
| Underrepresented | 1,722,766 (11.6%) | 430,542 (8.9%) | 1,292,224 (12.8%) |
|  |  |  |  |
| Dual-Eligible; n(%) | 3,357,707 (22.5%) | 1,494,664 (30.9%) | 1,863,043 (18.5%) |
| Original Reason for Medicare; n(%) |  |  |  |
| Age | 11,082,601 (74.40%) | 3,127,031 (64.72%) | 7,955,570 (79.04%) |
| Disability | 3,799,444 (25.51%) | 1,699,559 (35.18%) | 2,099,885 (20.86%) |
| ESRD | 6,088 (0.04%) | 1,398 (0.03%) | 4,690 (0.05%) |
| Disability and ESRD | 8,482 (0.06%) | 3,618 (0.07%) | 4,864 (0.05%) |
|  |  |  |  |
| Plan Type |  |  |  |
| HMO | 8,979,510 (60.3%) | 2,398,642 (49.6%) | 6,580,868 (65.4%) |
| PPO | 5,711,179 (38.3%) | 2,360,175 (48.8%) | 3,351,004 (33.3%) |
| Other | 205,926 (1.4%) | 72,789 (1.5%) | 133,137 (1.3%) |
| Rural; n(%) | 2,099,758 (14.1%) | 927,567 (19.2%) | 1,172,191 (11.6%) |
|  |  |  |  |
| CMS-HCC Risk Score; Mean [SD]^*^ | 1.194 [1.057] | 1.277 [1.107] | 1.154 [1.031] |
| Decedent Status; n(%) |  |  |  |
| Decedent | 316,389 (2.1%) | 127,057 (2.6%) | 189,332 (1.9%) |
| Near-Decedent | 612,747 (4.1%) | 244,325 (5.1%) | 368,422 (3.7%) |
| Non-Decedent | 13,967,479 (93.8%) | 4,460,224 (92.3%) | 9,507,255 (94.5%) |
|  |  |  |  |
|  |  |  |  |

^*^ All MA risk scores expressed prior to CMS 5.9% Coding Intensity Factor adjustment.

**MA Population Characteristics, 2020**

|  | Overall MA Population | Switchers | Non-Switchers |
| --- | --- | --- | --- |
| # of Beneficiaries | 1,183,074 | 377,797 | 805,277 |
| # of Beneficiary Months | N = 13,619,479 | n = 4,226,627 | n = 9,392,852 |
| Female; n(%) | 7,704,932 (56.6%) | 2,354,257 (55.7%) | 5,350,675 (57.0%) |
| Age; n(%) |  |  |  |
| <65 | 1,763,716 (12.9%) | 754,873 (17.9%) | 1,008,843 (10.7%) |
| 65-74 | 6,124,888 (45.0%) | 1,294,306 (30.6%) | 4,830,582 (51.4%) |
| 75-84 | 4,208,347 (30.9%) | 1,594,906 (37.7%) | 2,613,441 (27.8%) |
| 85+ | 1,522,528 (11.2%) | 582,542 (13.8%) | 939,986 (10.0%) |
| Race; n(%) |  |  |  |
| Black | 1,788,680 (13.1%) | 672,024 (15.9%) | 1,116,656 (11.9%) |
| White | 10,292,974 (75.6%) | 3,178,371 (75.2%) | 7,114,603 (75.7%) |
| Underrepresented | 1,537,825 (11.3%) | 376,232 (8.9%) | 1,161,593 (12.4%) |
|  |  |  |  |
| Dual-Eligible; n(%) | 2,904,085 (21.3%) | 1,267,025 (30.0%) | 1,637,060 (17.4%) |
| Original Reason for Medicare; n(%) |  |  |  |
| Age | 10,168,858 (74.66%) | 2,757,389 (65.24%) | 7,411,469 (78.91%) |
| Disability | 3,437,974 (25.24%) | 1,465,314 (34.67%) | 1,972,660 (21.00%) |
| ESRD | 5,105 (0.04%) | 1,020 (0.02%) | 4,085 (0.04%) |
| Disability and ESRD | 7,542 (0.06%) | 2,904 (0.07%) | 4,638 (0.05%) |
|  |  |  |  |
| Plan Type |  |  |  |
| HMO | 8,369,630 (61.5%) | 2,111,058 (49.9%) | 6,258,572 (66.6%) |
| PPO | 5,023,554 (36.9%) | 2,040,414 (48.3%) | 2,983,140 (31.8%) |
| Other | 226,295 (1.7%) | 75,155 (1.8%) | 151,140 (1.6%) |
| Rural; n(%) | 1,831,855 (13.5%) | 769,635 (18.2%) | 1,062,220 (11.3%) |
|  |  |  |  |
| CMS-HCC Risk Score; Mean [SD]^*^ | 1.250 [1.090] | 1.340 [1.149] | 1.209 [1.061] |
| Decedent Status; n(%) |  |  |  |
| Decedent | 298,665 (2.2%) | 115,444 (2.7%) | 183,221 (2.0%) |
| Near-Decedent | 591,975 (4.3%) | 224,558 (5.3%) | 367,417 (3.9%) |
| Non-Decedent | 12,728,839 (93.5%) | 3,886,625 (92.0%) | 8,842,214 (94.1%) |
|  |  |  |  |
|  |  |  |  |

^*^ All MA risk scores expressed prior to CMS 5.9% Coding Intensity Factor adjustment.

**Geographic Variation: State-Level Sample Sizes**

|  | 2022 | | 2021 | | 2020 | |
| --- | --- | --- | --- | --- | --- | --- |
| State | Member Months (N) | Percent of Study Population (%) | Member Months (N) | Percent of Study Population (%) | Member Months (N) | Percent of Study Population (%) |
| AK | 1,525 | 0.0% | 1,378 | 0.0% | 1,154 | 0.0% |
| AL | 317,746 | 2.0% | 292,807 | 2.0% | 263,193 | 1.9% |
| AR | 136,265 | 0.8% | 121,828 | 0.8% | 105,762 | 0.8% |
| AZ | 368,491 | 2.3% | 343,663 | 2.3% | 316,990 | 2.3% |
| CA | 1,730,060 | 10.7% | 1,644,402 | 11.0% | 1,563,113 | 11.5% |
| CO | 258,434 | 1.6% | 237,962 | 1.6% | 222,342 | 1.6% |
| CT | 197,999 | 1.2% | 181,323 | 1.2% | 166,710 | 1.2% |
| DC | 13,845 | 0.1% | 12,714 | 0.1% | 11,204 | 0.1% |
| DE | 34,909 | 0.2% | 29,055 | 0.2% | 22,965 | 0.2% |
| FL | 1,407,193 | 8.7% | 1,321,553 | 8.9% | 1,230,989 | 9.0% |
| GA | 502,478 | 3.1% | 459,409 | 3.1% | 412,778 | 3.0% |
| HI | 81,234 | 0.5% | 76,263 | 0.5% | 71,994 | 0.5% |
| IA | 115,394 | 0.7% | 101,654 | 0.7% | 89,547 | 0.7% |
| ID | 87,689 | 0.5% | 79,148 | 0.5% | 69,993 | 0.5% |
| IL | 476,103 | 2.9% | 420,496 | 2.8% | 373,101 | 2.7% |
| IN | 316,565 | 2.0% | 288,259 | 1.9% | 252,277 | 1.9% |
| KS | 86,844 | 0.5% | 74,754 | 0.5% | 62,383 | 0.5% |
| KY | 253,067 | 1.6% | 227,093 | 1.5% | 199,173 | 1.5% |
| LA | 246,156 | 1.5% | 225,692 | 1.5% | 201,276 | 1.5% |
| MA | 242,019 | 1.5% | 219,144 | 1.5% | 199,931 | 1.5% |
| MD | 113,092 | 0.7% | 95,817 | 0.6% | 75,741 | 0.6% |
| ME | 101,854 | 0.6% | 91,790 | 0.6% | 77,936 | 0.6% |
| MI | 657,422 | 4.1% | 604,615 | 4.1% | 548,700 | 4.0% |
| MN | 325,052 | 2.0% | 301,093 | 2.0% | 273,736 | 2.0% |
| MO | 328,531 | 2.0% | 302,197 | 2.0% | 271,558 | 2.0% |
| MS | 117,487 | 0.7% | 99,110 | 0.7% | 79,395 | 0.6% |
| MT | 34,899 | 0.2% | 31,189 | 0.2% | 27,079 | 0.2% |
| NC | 561,692 | 3.5% | 505,950 | 3.4% | 451,307 | 3.3% |
| ND | 21,117 | 0.1% | 17,425 | 0.1% | 15,104 | 0.1% |
| NE | 57,276 | 0.4% | 47,381 | 0.3% | 39,437 | 0.3% |
| NH | 51,187 | 0.3% | 43,287 | 0.3% | 36,102 | 0.3% |
| NJ | 341,500 | 2.1% | 315,370 | 2.1% | 286,927 | 2.1% |
| NM | 112,921 | 0.7% | 103,582 | 0.7% | 95,060 | 0.7% |
| NV | 142,800 | 0.9% | 130,881 | 0.9% | 118,143 | 0.9% |
| NY | 981,488 | 6.1% | 922,846 | 6.2% | 869,759 | 6.4% |
| OH | 683,739 | 4.2% | 637,353 | 4.3% | 595,481 | 4.4% |
| OK | 145,800 | 0.9% | 126,703 | 0.9% | 102,975 | 0.8% |
| OR | 259,193 | 1.6% | 242,791 | 1.6% | 229,746 | 1.7% |
| PA | 762,405 | 4.7% | 717,222 | 4.8% | 678,916 | 5.0% |
| RI | 69,359 | 0.4% | 63,689 | 0.4% | 58,956 | 0.4% |
| SC | 256,207 | 1.6% | 228,819 | 1.5% | 201,633 | 1.5% |
| SD | 30,853 | 0.2% | 26,359 | 0.2% | 23,402 | 0.2% |
| TN | 377,813 | 2.3% | 351,496 | 2.4% | 324,944 | 2.4% |
| TX | 1,176,835 | 7.3% | 1,083,536 | 7.3% | 978,356 | 7.2% |
| UT | 108,598 | 0.7% | 98,475 | 0.7% | 89,668 | 0.7% |
| VA | 286,988 | 1.8% | 248,359 | 1.7% | 212,584 | 1.6% |
| VT | 23,326 | 0.1% | 16,513 | 0.1% | 11,726 | 0.1% |
| WA | 332,729 | 2.1% | 303,060 | 2.0% | 274,024 | 2.0% |
| WI | 348,981 | 2.2% | 322,430 | 2.2% | 298,042 | 2.2% |
| WV | 115,689 | 0.7% | 105,569 | 0.7% | 94,635 | 0.7% |
| WY | 5,640 | 0.0% | 3,796 | 0.0% | 3,179 | 0.0% |
| Other* | 356,552 | 2.2% | 349,315 | 2.3% | 338,353 | 2.5% |
| Total | **16,163,041** | **100.0%** | **14,896,615** | **100.0%** | **13,619,479** | **100.0%** |

*U.S. Territories / Unknown

***Comparison of MA Switchers in Medicare Limited Data Set Standard Analytical Files (Five Percent Sample) and MA Encounter Data***

In our attempt to replicate MedPAC’s estimate of favorable selection for TM-to-MA switchers, we used the Medicare Limited Data Set Standard (LDS) Analytical Files from a five percent random sample of Medicare beneficiaries from January 1, 2008 to December 31, 2022. We identified annual cohorts of beneficiaries who switched from TM to MA between January 1, 2010 and December 31, 2022. We used LDS claims from January 1, 2009 through December 31, 2021 to measure pre-switch cost patterns. Beneficiaries were required to have at least two full years of continuous enrollment in TM Parts A and B prior to MA enrollment. Eligible beneficiaries were assigned to a reference year, defined as the year before switching to MA. Beneficiaries were excluded on a monthly basis from the study if they did not have Parts A and B coverage, were enrolled in hospice, were eligible for Medicare due to end-stage renal disease status, had Medicare as a secondary payer, or lived outside of the 50 United States or Washington, DC. Then, to extrapolate findings from switchers to all MA enrollees, we used MA encounter data from January 1, 2020 to December 31, 2022 for all MA beneficiaries, including those identified as switchers (using the same criteria as described above).

Because switchers were identified separately in the LDS and MA encounter data, the samples may not comprise identical individuals. To the best of our knowledge, switchers largely comprised of the same underlying beneficiaries in both the LDS and MA encounter data. CMS defines the five percent enhanced sample based on fixed digits of the beneficiary’s Health Insurance Claim (HIC) number, which includes beneficiaries whose Claim Account Number ends in one of five predetermined digit pairs (05, 20, 45, 70, or 95); because HIC digits are stable over time, this sampling mechanism yields a relatively consistent cohort of enrollees in both files. Because switchers are identified separately in the LDS and MA encounter data, we assessed sample comparability by comparing descriptive characteristics. The MA encounter data included approximately 2.5% fewer individuals, but demographic characteristics were largely similar across samples. The comparison between our two switcher populations is shown below:

**MA-to-TM Switchers: Comparison of Population Characteristics in CMS Limited Data Set vs. MA Encounter Data, 2022**

|  | LDS 5% Sample | MA Encounter Data |
| --- | --- | --- |
| # of Beneficiaries | 497,720 | 475,813 |
| # of Beneficiary Months | n = 5,476,633 | n = 5,340,368 |
| Female; n(%) | 3,043,005 (55.6%) | 2,965,794 (55.5%) |
| Age; n(%) |  |  |
| <65 | 955,785 (17.5%) | 937,071 (17.5%) |
| 65-74 | 1,640,550 (30.0%) | 1,601,347 (30.0%) |
| 75-84 | 2,114,356 (38.6%) | 2,062,556 (38.6%) |
| 85+ | 765,942 (14.0%) | 739,394 (13.8%) |
| Race; n(%) |  |  |
| Black | 869,023 (15.9%) | 846,869 (15.9%) |
| White | 4,109,859 (75.0%) | 4,005,198 (75.0%) |
| Underrepresented | 497,751 (9.1%) | 488,301 (9.1%) |
|  |  |  |
| Dual-Eligible; n(%) | 1,743,014 (31.8%) | 1,709,224 (32.0%) |
| Original Reason for Medicare; n(%) |  |  |
| Age | 3,517,814 (64.2%) | 3,428,425 (64.2%) |
| Disability | 1,953,377 (35.7%) | 1,906,690 (35.7%) |
| ESRD | 1,554 (0.0%) | 1,505 (0.0%) |
| Disability and ESRD | 3,888 (0.1%) | 3,748 (0.1%) |
|  |  |  |
| Rural; n(%) | 1,090,112 (19.9%) | 1,070,222 (20.0%) |
|  |  |  |
| Decedent Status; n(%) |  |  |
| Decedent | 134,154 (2.4%) | 129,928 (2.4%) |
| Near-Decedent | 263,667 (4.8%) | 255,495 (4.8%) |
| Non-Decedent | 5,078,812 (92.7%) | 4,954,945 (92.8%) |

**Section 2: Cost Imputation**

MA encounter data does not contain price information available for research, only utilization. In this analysis, we built on the approach developed by Jung et al., 2022 to apply the traditional Medicare (TM) allowed cost (based on the fee-for-service (FFS) benefit structure) to the data to allow for cost comparisons between MA and TM populations. This approach accounts for service type and severity, as well as state-level cost variation. We enhanced this methodology by incorporating more granular TM data (e.g., beneficiary county, national provider identifier) and making relevant payment adjustments, including Healthcare Common Procedure Coding System (HCPCS) modifiers and outlier payments, to yield more precise cost estimates. We also broadened the scope to include skilled nursing facility (SNF) and Home Health service categories.

***Cost Imputation Methodology Overview***


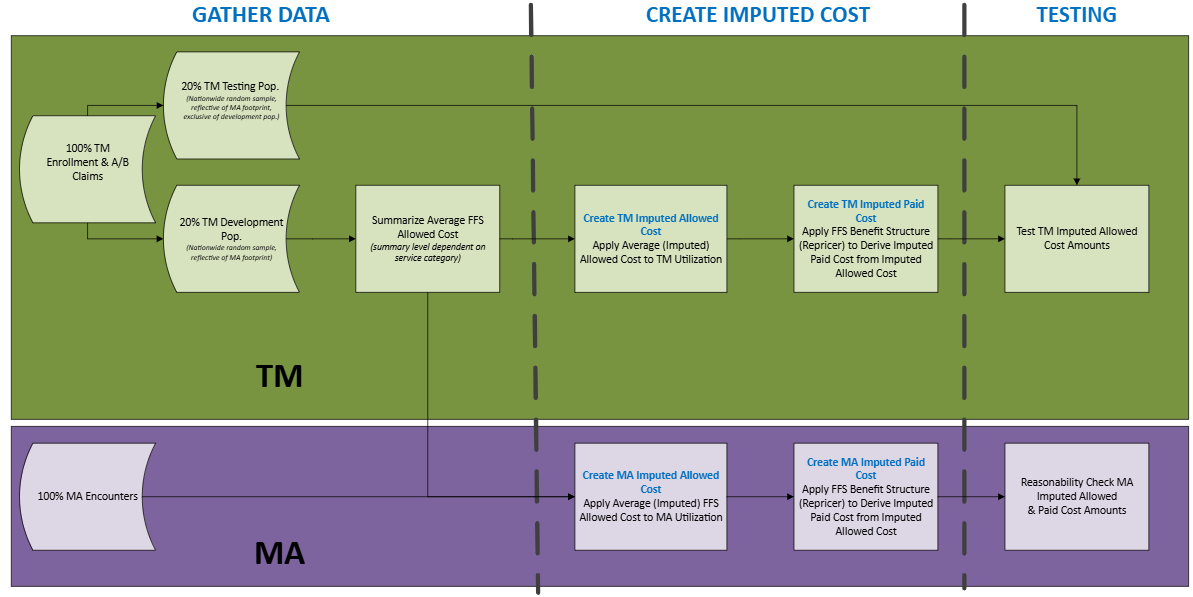


We calculated the imputed TM cost for each service category, including inpatient, outpatient, carrier, durable medical equipment (DME), skilled nursing facility (SNF), and home health. We included Part A and Part B MA encounter data, excluding services not covered in TM (e.g., chart reviews, supplemental benefits, etc.).

We first estimated the allowed amounts for each service category, with methodologies detailed below.

| Service Category | Cost Imputation Methodology |
| --- | --- |
| Inpatient | - Allowed cost per admission based on published weights by Diagnosis Related Group (DRG) code^1^. TM Data is aggregated by DRG code, Organization National Provider Identifier (NPI), beneficiary county, beneficiary state, and outlier cost binary flag^2^. Standardized prices are then applied to the MA encounter data by group. |
| Outpatient | - Allowed cost per claim line based on the average actual TM cost by Healthcare Common Procedure Coding System (HCPCS) code, attending physician index, Organization NPI, beneficiary county, and beneficiary state. Standardized prices are then applied to the MA encounter data by group. |
| Carrier | - Allowed cost per unit of service based on the published Resourced-Based Relative Value Schedule (RBRVS) by HCPCS code^3^ for Professional Services; Laboratory and Part B prescription drug claims based on their own published fee schedules. TM data is aggregated by HCPCS code, HCPCS modifier code, beneficiary county, and beneficiary state. Standardized prices are then applied to the MA encounter data by group. |
| DME | - Allowed cost per unit of service based on the published fee schedule by HCPCS code^4^. TM data is aggregated by HCPCS code, HCPCS modifier code, beneficiary county, and beneficiary state. Standardized prices are then applied to the MA encounter data by group. |
| SNF | - Developed unique logic to price MA encounters using FFS payment methodology (i.e., applied Resource Utilization Groups Version IV (RUG-IV) rates prior to 10/1/2019 and Patient-Driven Payment Model (PDPM) rates after 10/1/2019). |
| Home Health | - Developed unique logic to price MA encounters using FFS payment methodology (i.e., applied Patient Driven Groupings Model rates to claims after 1/1/2020 and Home Health Resource Group prior to 1/1/2020). |

^1^ https://www.cms.gov/medicare/payment/prospective-payment-systems/acute-inpatient-pps

^2^Outlier cost flag based on claims where the Claim Related Condition Code = ‘61’.

^3^ https://www.cms.gov/medicare/payment/fee-schedules/physician/pfs-relative-value-files

^4^ https://www.cms.gov/medicare/payment/fee-schedules/dmepos/dmepos-fee-schedule

We matched the TM population distribution to the MA footprint at the state level. We then applied the FFS costs to MA iteratively for each service category. Using the inpatient service category as an illustrative example, we performed the following steps: (1) aimed to match by DRG code, outlier flag, and organization NPI, (2) if there was no match, we looked for a match on DRG code, outlier flag, and beneficiary county, (3) if there was no match, we then looked for a match on DRG code, outlier flag, and beneficiary state, and (4) lastly, if there was no match, we matched on DRG code and outlier flag.

After applying the FFS allowed amounts onto the encounter data, we then applied the FFS cost sharing to derive payment amounts, using the following methodology: (1) we summarized the allowed claims by Part A vs. Part B for each calendar year, (2) we sorted data by beneficiary and claim date, (3) we calculated Part A/B deductibles and Part A/B cost sharing on each claim, based on FFS benefit structure for the given calendar year (4) we removed cost sharing amount from the allowed amount for each claim to derive the paid amount, and (5) we applied sequestration to the paid amount. The result reflects what FFS Medicare would have paid for the claim had the beneficiary been enrolled in TM.

***Goodness-of-Fit for Cost Imputation Methodology***

Since MA encounter data does not have any plan-submitted cost amounts available for study, we were unable to directly test the accuracy of our imputed cost methodology using MA encounter data. Instead, to assess the accuracy of our methods on the TM population, we compared the imputed allowed amounts against the actual allowed amounts for TM. We computed the difference at a member level for 2017 through 2022, grouping members into percentiles based on the variance. We found that nearly 70% (69.4%) of imputed claims were within 5% of the actual allowed amount in 2022, with comparable performance in earlier years.

**Member-Level Absolute Variance in Imputed Allowed Costs vs. Actual Allowed Costs for TM, 2017-2022**

| **Cumulative Distribution** | **2017** | **2018** | **2019** | **2020** | **2021** | **2022** |
| --- | --- | --- | --- | --- | --- | --- |
| Absolute % Variance <= 2% | 42.0% | 42.4% | 42.2% | 41.7% | 41.7% | 41.9% |
| Absolute % Variance Between 2% and 5% | 69.5% | 69.6% | 68.8% | 69.2% | 68.9% | 69.4% |
| Absolute % Variance Between 5% and 10% | 84.9% | 85.0% | 84.4% | 85.0% | 84.6% | 84.8% |
| Absolute % Variance Between 10% and 20% | 93.9% | 93.9% | 93.6% | 93.9% | 93.6% | 93.7% |
| Absolute % Variance Between 20% and 30% | 96.6% | 96.6% | 96.4% | 96.5% | 96.5% | 96.4% |
| Absolute % Variance Between 30% and 40% | 97.9% | 97.9% | 97.7% | 97.8% | 97.9% | 97.7% |
| Absolute % Variance Between 40% and 50% | 98.7% | 98.7% | 98.5% | 98.6% | 98.6% | 98.5% |
| Absolute % Variance Between 50% and 75% | 99.7% | 99.7% | 99.6% | 99.6% | 99.5% | 99.5% |
| Absolute % Variance Between 75% and 100% | 100.0% | 100.0% | 99.9% | 99.9% | 99.8% | 99.8% |
| Absolute % Variance > 100% | 100.0% | 100.0% | 100.0% | 100.0% | 100.0% | 100.0% |

Because our extrapolation approach compared the relative difference in average per-member -per-month (PMPM) costs between switchers and non-switchers, the relevant metric is the percentage difference in mean imputed versus actual costs at the population level, not at the member level. The large size of both groups ensures that any member-level imputation error is minimized by the law of large numbers. On average, imputed and actual allowed costs differed by 0.2%, with over- and under-predictions distributed evenly. Further, all service categories saw an improvement in the distribution of the variance compared to Jung et al., 2022. For purposes of comparing imputed costs between switchers and non-switchers, we assumed that our imputation methodology predicts cost with equal accuracy for both switchers and non-switchers, allowing an unbiased evaluation of relative costs.

**Average Percent Difference in Actual vs. Allowed Costs in TM, 2017-2022**

| **Year** | **Actual Allowed Cost** | **Imputed Allowed Cost** | **Difference in Actual vs. Allowed Cost** | **Average % Difference in Actual vs. Allowed Cost** |
| --- | --- | --- | --- | --- |
| **2017** | $16,628,861,238 | $16,648,482,667 | $19,621,429 | 0.10% |
| **2018** | $17,109,896,935 | $17,128,769,806 | $18,872,871 | 0.10% |
| **2019** | $17,694,697,072 | $17,734,764,949 | $40,067,877 | 0.20% |
| **2020** | $16,363,626,643 | $16,355,473,458 | -$8,153,184 | 0.00% |
| **2021** | $16,904,246,964 | $16,947,504,478 | $43,257,514 | 0.30% |
| **2022** | $16,906,671,904 | $16,942,014,604 | $35,342,701 | 0.20% |

To compare this study’s imputed cost methodology to Jung et al., 2022, we calculated predicted minus actual allowed cost for each member's claims within a service category to get the prediction error.  We rank ordered the magnitude of the prediction error at the member level, then grouped by percentiles. We then summarized the average prediction error within each percentile group:

**Comparison of Percent Difference in FFS Actual vs. Predicted Allowed Cost, Jung et al. (2022) vs. This Study’s Imputed Cost Methodology by Service Category**

***Inpatient:***

**
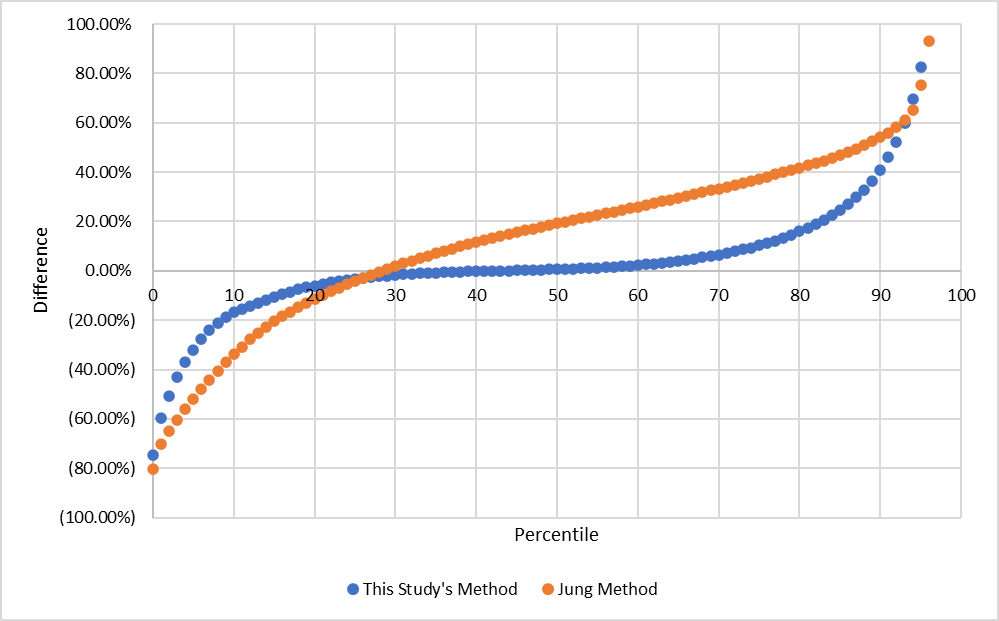
**

***Outpatient:***

***
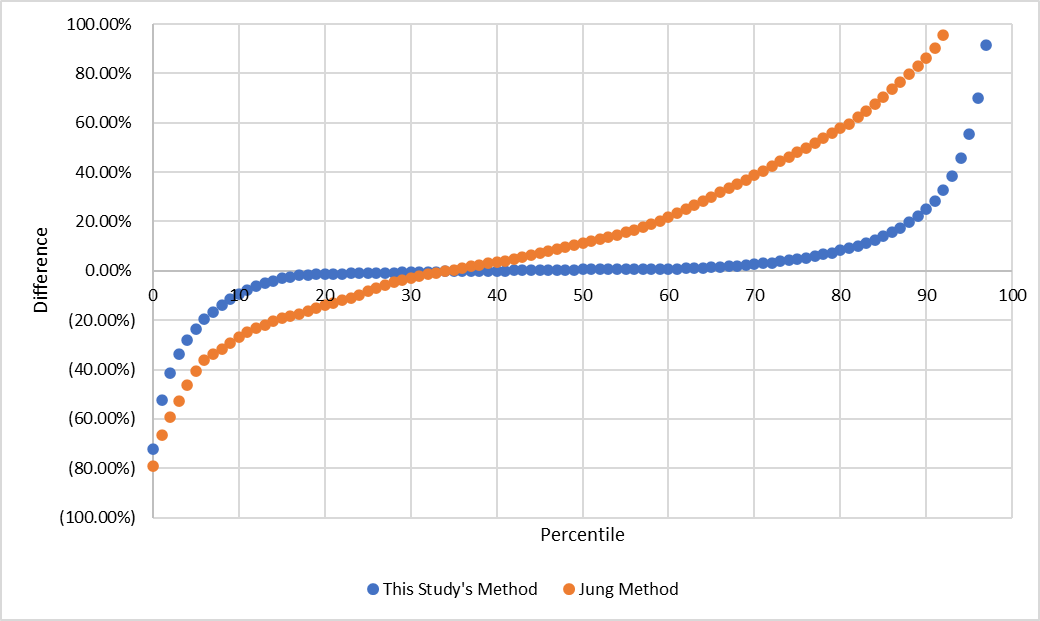
***

***DME:***

***
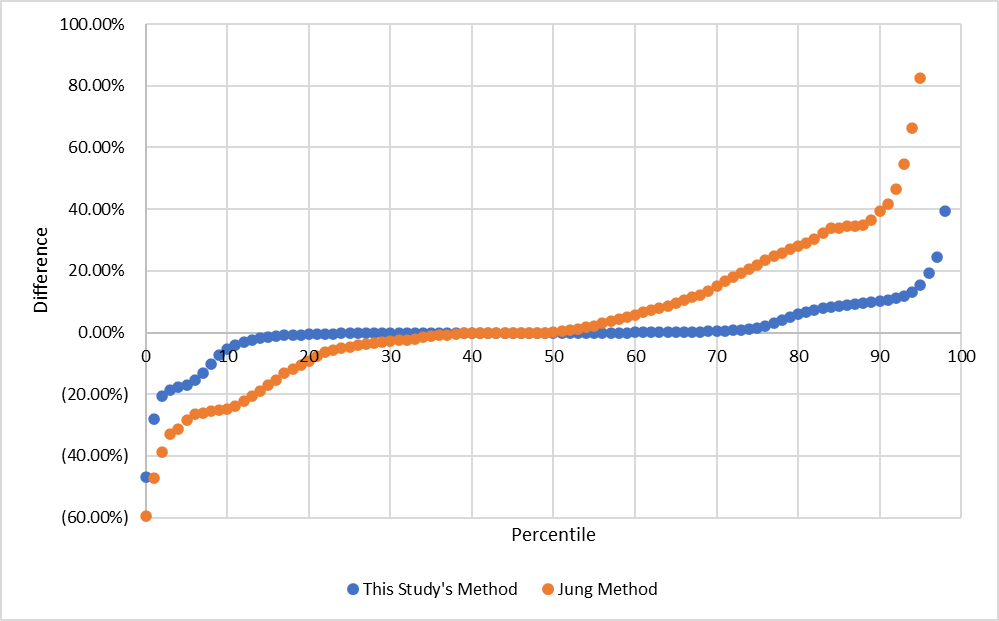
***

***Carrier:***

***
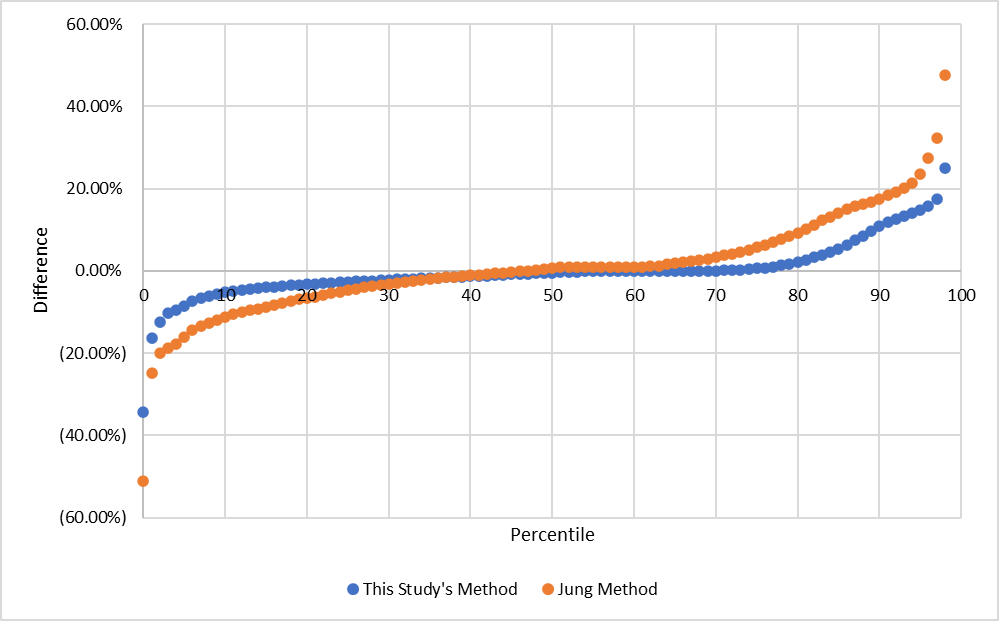
***

**Section 3: Methodology for Estimating Favorable Selection Among TM-to-MA Switchers**

To estimate favorable selection among beneficiaries who switched from TM to MA, we followed the methodology described in MedPAC’s March 2025 report. We compared pre-switch risk-adjusted monthly spending between TM-to-MA switchers and beneficiaries who remained in TM, with separate estimates for each cohort of MA switchers by payment year. Risk-adjusted spending was calculated by dividing per-member per-month paid costs for Medicare Parts A and B by the average CMS-HCC risk score within each population. We then measured regression-to-the-mean within each cohort to capture the extent to which favorable selection converged towards average levels over time, using MedPAC’s methodology. We did this by matching each switcher cohort to a TM “proxy” group, selected to mirror (1) the distribution of base year selection percentages for each switcher cohort, and (2) mortality by year. We then stratified by selection percentages, and projected future-year selection percentages among switchers based on the observed pattern within the matched TM group.

Our estimates may differ from MedPAC’s due to methodological differences, such as data constraints and gaps in the explanation of the methods in the MedPAC report. With respect to the data, we only had access to the 5% Limited Data Set (LDS) going back to 2008, which is two fewer years of lookback period than MedPAC’s analysis. Further, MedPAC’s analysis incorporated the 100% Innovator Data Set. The impact of this difference is that we identified 33.9% of MA members as switchers (as opposed to 33.0% in MedPAC’s analysis). In addition, for 2008 and 2009, the LDS hospice data include only quarterly claims, preventing us from identifying the exact months of hospice enrollment. As a result, hospice months are likely overidentified, inflating estimated base spending relative to TM for MA entrants in 2010. This occurs because spending in the months leading up to hospice enrollment is typically high, and overrepresenting these months raises the estimated baseline. While this issue affects a small share of switchers (3.4%), the 2010 cohort represents the oldest group and is used to extrapolate to 26.6% of non‑switchers in 2021, potentially amplifying its influence on estimated favorable selection.

While we sought to replicate MedPAC’s methods as closely as possible, several methodological differences were necessary due to data constraints. The MedPAC report does not specify how beneficiaries with Medicare as a secondary payer were identified. In the 5% LDS, we could identify such beneficiaries only when a TM claim reflected payment from a non‑Medicare payer; MedPAC may have had access to additional CMS data that enabled more complete identification. As a result, our approach may under‑identify beneficiaries with Medicare as secondary coverage, potentially increasing the size of the switcher cohort relative to what would be observed with more comprehensive data. Data limitations also limited replication of MedPAC’s county‑level matching to control for geographic differences between TM stayers and MA switchers. Given credibility concerns associated with conducting county‑level analyses in the 5% sample, we instead controlled for area differences using the 2020-2022 CMS Ratebook, which CMS uses to capture county‑level variation in risk‑adjusted traditional Medicare costs.

In addition, while MedPAC provides substantial methodological detail, certain implementation specifics were not documented and required interpretation. For example, the report does not describe how cutoffs for the actual‑to‑expected risk transfer margin bins were defined to measure regression-to-the-mean, nor how beneficiaries who switched multiple times between MA and TM were classified across cohorts. Similar ambiguities may exist in other aspects of beneficiary classification or calculation methods, creating additional opportunities for divergence from MedPAC’s approach. A methodological comparison between our approach and MedPAC’s is detailed below:

**Methodological Comparison Between the Present Study vs. the MedPAC 2025 Report**

| **Analysis Component** | **MedPAC (March 2025)** | **This Study** |
| --- | --- | --- |
| Data Set | 100% TM data from the Innovator data set | 5% TM sample from the Limited data set |
| Study Period | 2006-2022 | 2008-2022 |
| Study Sample | TM-to-MA Switchers from 2008-2022 | TM-to-MA Switchers from 2010-2022 |
| Inclusion Criteria | Two years of continuous enrollment in Part A and B prior to MA enrollment | Same as MedPAC’s March 2025 Report |
| Exclusion Criteria | Excluded for months without Parts A and B coverage, in hospice, eligible for Medicare due to end-stage renal disease, had Medicare as a secondary payer, or lived outside of the 50 U.S. states or Washington, DC. | Same as MedPAC’s March 2025 Report |
| Initial Selection | Compares pre-switch costs between TM-to-MA switchers, compared to those remaining in TM | Same as MedPAC’s March 2025 Report |
| Regression-to-the-Mean | Assumes switchers’ selection percentages approach toward mean at same rate as proxy cohort of those remaining in TM | Same as MedPAC’s March 2025 Report |
| Accounting for geographic variation | Matched MA switchers with TM stayers at the county-level. Aggregate results using switchers’ county enrollment as weight. | Used the 2020-2022 CMS Ratebook, which measures county-level differences in risk adjusted TM costs |

We compare our results to the methodology used in MedPAC’s March 2025 Report. However, MedPAC’s approach has continued to evolve. In its March 2026 Report, MedPAC made the following changes to their analysis of switchers: (1) the incorporation of end-stage renal disease beneficiaries; (2) refinement of proxy group matching using dual eligibility status (fully dual, partially dual, and non‑dual); (3) removal of weighting for hospice months; (4) restrictions to ensure consistent county of residence between the MA switcher population and TM comparison group; and (5) corrections to prior estimates related to the treatment of decedent months, including adjustments affecting the 2016 estimate and earlier years reported in the March 2025 report.

We also present diagnostics to better understand where our attempt to replicate MedPAC’s 2025 approach diverges from their published result. First, we display risk-adjusted spending for TM-MA switchers vs. TM stayers in the year prior to switching to inform whether selection effects differs from MedPAC at the time of switching versus in subsequent years. MedPAC did not report estimates separately for switchers and non-switchers, and therefore, we cannot disentangle whether our estimate differs from MedPAC’s in the study population, the non-study population, or both. However we were able to closely replicate MedPAC’s risk-adjusted spending for TM-MA switchers vs. TM stayers in the year prior to beneficiary MA entry. Shown below is a version of Figure 11-7 from MedPAC’s 2025 report, showing that our calculation produces results very close to MedPAC’s.

**This Study’s Comparison of Risk-Adjusted Spending for TM-MA Switchers vs. TM Stayers In Year Before MA Entry:**


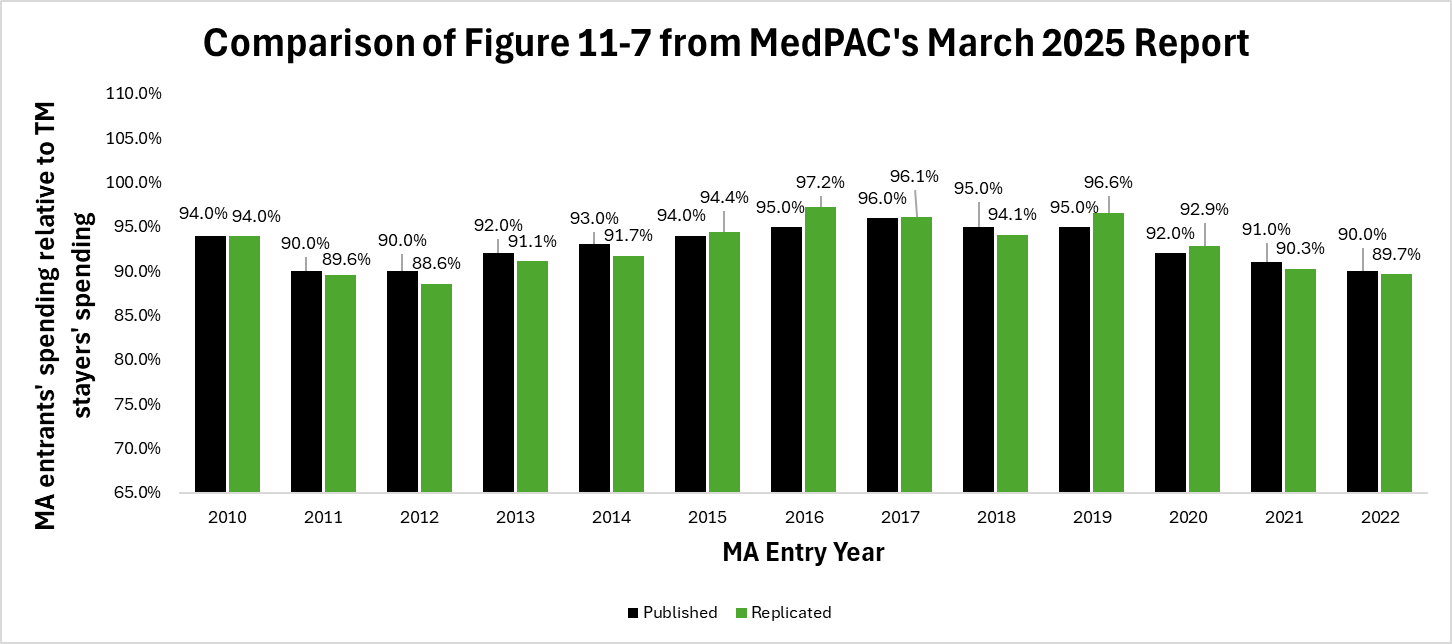


We also replicate Figure 11-A1 from MedPAC’s 2025 Report to examine reversion to the mean annually for switchers – specifically, how favorable selection evolves with years since switching – to highlight how favorable selection changes the longer beneficiaries are enrolled in MA. It is important to note that MedPAC’s 2025 Table 11-A1 does not separate estimates for mean reversion versus extrapolation. Instead, MedPAC incorporates the impact of extrapolation directly in its measures, “change in selection percentage while in MA” and “selection percentage trended forward to 2021,” which precludes a direct comparison of selection dynamics among switchers by duration of MA enrollment between our study and MedPAC’s. Several key details required to conduct this analysis separately for switchers and non-switchers are not provided in MedPAC’s appendix.

Given this limitation, we replicated Table 11-A1 as closely as possible, including the effects of extrapolation, to ensure an apples-to-apples comparison between our study and MedPAC’s published estimates. We have included this table below. This approach represents the most direct and transparent comparison feasible given the structure of MedPAC’s reported results. Our replication of Table 11-A1 indicates challenges in reproducing MedPAC’s initial selection calculation, as shown by the largest discrepancy observed in base spending relative to TM. Because we closely replicated MedPAC’s estimates for MA entrants’ spending relative to TM stayers, the discrepancies likely arise in later steps: (1) including decedents and switchers in the denominator, (2) restricting the numerator to individuals surviving to 2021, and/or (3) weighting member months using the reference year rather than the subsequent year.


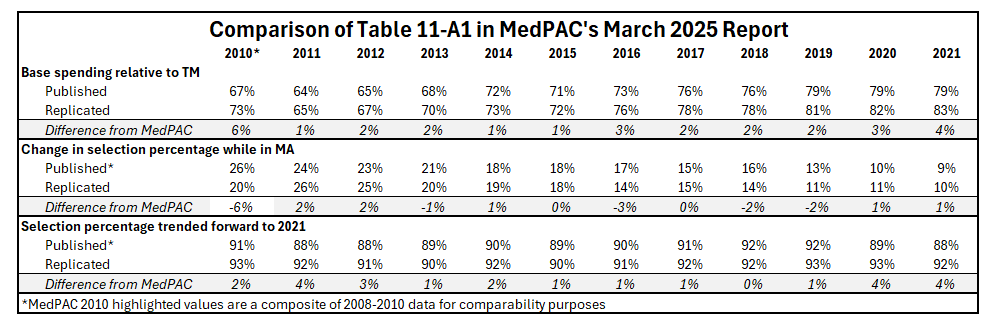


**Section 4: Sensitivity Analyses**

*MA Exposure Effect*

Because this study uses MA encounter data, differences in risk-adjusted spending and risk scores between switchers and non-switchers reflect both favorable selection and post-enrollment plan-driven effects, including coding intensity, utilization management, provider networks. To address these factors, this study’s extrapolation approach accounts for differences in plan mix and geographic variation between switchers and non-switchers, with an additional adjustment for coding intensity. However, these adjustments may not fully account for all plan‑driven differences in risk‑standardized spending between switchers and non‑switchers, particularly if such effects trend differently between switchers and non-switchers. To assess sensitivity to these remaining uncertainties, we conducted extensive supplemental analyses, including tests that evaluate whether plan mix adjustments adequately capture post-enrollment plan effects by applying an “MA exposure effect,” examining how alternative values for switchers and non-switchers impacted our estimate.

To calculate the MA exposure effect, we rely on the premise that risk‑adjusted spending for MA switchers can be estimated using two data sources: TM claims (i.e., based on the counterfactual TM cost and risk score, as calculated using MedPAC’s methodology) and MA encounter data. Although the switcher populations may differ slightly across these sources, demographic characteristics suggest they are largely comparable (see Table “MA-to-TM Switchers: Comparison of Population Characteristics in CMS Limited Data Set vs. MA Encounter Data, 2022” in Section 1). We first estimate TM counterfactual costs and risk scores for switchers to obtain risk‑standardized spending. We then compare per‑member‑per‑month (PMPM) costs and risk scores for the same group using MA encounter data. The MA exposure effect is defined as the ratio of encounter‑based to TM‑based PMPM costs and risk scores, calculated separately for each measure. Next, we adjust the non-switchers’ encounter data for geography and plan mix to ensure that non-switchers’ data is appropriately comparable to switchers’ encounter data. Then, taking the geography and plan adjusted PMPM costs and risk scores for non-switchers, we divide each respective measure by the MA exposure effect for switchers to derive the TM counterfactual costs for non-switchers. Finally, we assess the selection percentages for MA switchers, non-switchers, and the overall MA population. Detailed calculations are shown below:

**Step 1:** Estimate the TM counterfactual costs and risk score for switchers to obtain risk-standardized spending.

**Step 2:** Compare MA switchers’ per-member per-month (PMPM) costs and risk scores for approximately the same group of MA switchers using TM claims and MA encounter data. Calculate the MA exposure effect, defined as the ratio of encounter-based to TM-based PMPM costs and risk scores, calculated separately for each measure.

**Step 3:** Adjust non-switchers’ encounter data for geography and plan mix to ensure that non-switchers’ data is appropriately comparable to switchers’ encounter data.

**Step 4:** Taking the geography and plan-adjusted PMPM costs and risk scores for non-switchers, we divide each respective measure by the MA exposure effect for switchers to derive the TM counterfactual costs for non-switchers.

**Step 5:** Assess the selection percentages for MA switchers, non-switchers, and the overall MA population.


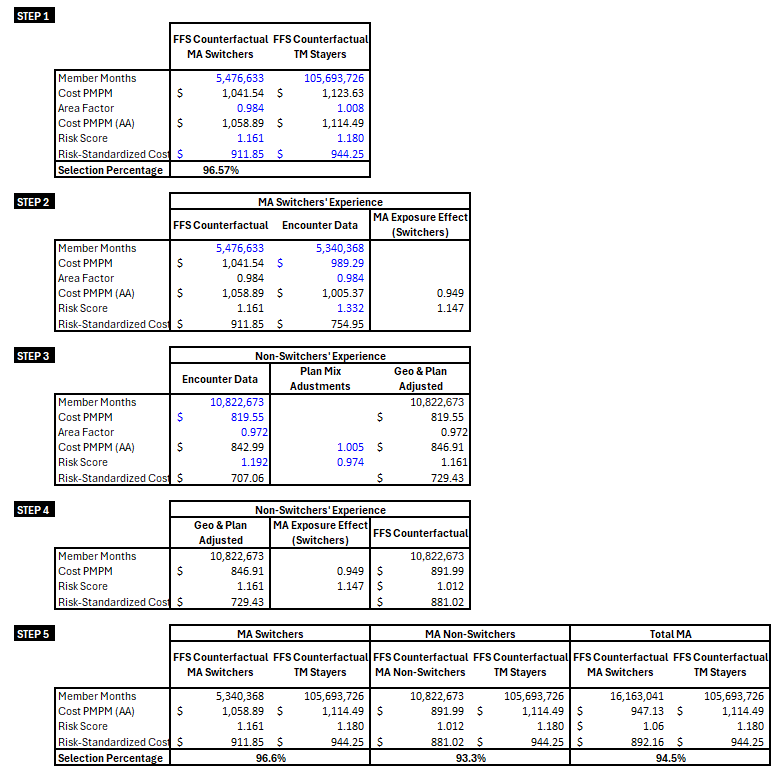


^*^ All MA risk scores expressed prior to CMS 5.9% Coding Intensity Factor adjustment.

We find that the MA exposure estimate for switchers’ medical spending reductions was 5.1% (i.e., MA exposure of 0.949) and coding was 7.9% (i.e., MA exposure of 1.079 after reducing MA risk scores by CMS’s adjustment of 5.9%^[[1]](#footnote-1)^). In our main analysis, we implicitly assume that these MA exposure estimates apply equally to the MA non-switcher population. However, we tested how small changes in MA exposure assumptions affect our favorable selection estimate. A 1 pp increase in claims exposure and a 2 pp increase in risk score exposure for switchers (relative to non-switchers) reduces the estimate by 0.6 pp. Conversely, a 1 pp increase in risk score exposure and a 2 pp increase in claims exposure raises the estimate by 1.3 pp, underscoring the sensitivity of our results to these assumptions.


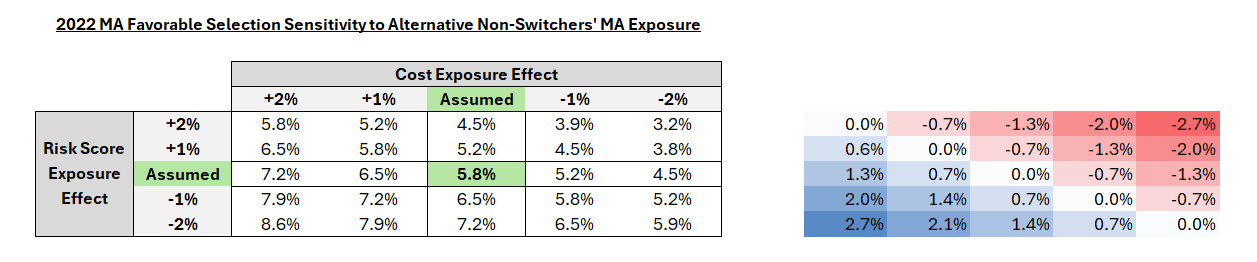


*Coding Intensity*

Our study relies on an assumption that MA risk scores are comparable between study and non-study populations, and that any differences in risk scores reflect population morbidity characteristics that would be present prior to enrollment in the MA plan. In calculating risk scores used for payment, CMS applies a uniform 5.9% reduction to MA risk scores^[[2]](#footnote-2)^, called the “MA Coding Pattern Difference Adjustment”, which we refer to as the “CIF” (coding intensity factor) below. The coding difference CMS intends to offset with this adjustment occurs in the HCC-based (diagnosis-based) portion of MA risk scores, and increases with MA duration (as demonstrated in MedPAC’s 2023 report^[[3]](#footnote-3)^).

In our main analysis, we assume a uniform coding intensity rate of 0% (effectively resulting in no coding differences when comparing MA switchers and non-switchers). Because uniform coding intensity adjustments across groups do not affect results; only relative differences between switchers and non-switchers matter, we tested sensitivity to differential coding between switchers and non-switchers. Since our extrapolation method compared risk-adjusted costs across these groups, differential coding practices could distort cost differences and bias the estimated magnitude of favorable selection. To test whether our estimate was sensitive to differences in coding intensity between switchers and non-switchers, we modeled different coding scenarios by applying the CIF non-uniformly. These tests were intended to better understand the extent to which coding differences between switchers and non-switchers might affect our analysis of favorable selection. Although our main analysis assumed comparable MA risk scores between switchers and non-switchers, we tested whether the following scenarios altered the result:

- Applying no CIF adjustment to the demographic portion of risk scores and no CIF adjustment to MA members in their first year after switching from TM (and varying the overall magnitude of the CIF applied)
- Applying a uniform CIF to switchers and non-switchers (e.g., CMS’s 5.9% CIF; estimated coding intensity from MedPAC’s 2025 report; 10.0%; and 15.0%)
- Applying no CIF adjustment to the demographic portion of risk scores, and no CIF adjustment to MA members in their first year after switching from TM.
- Applying a higher overall CIF adjustment to align with MedPAC’s 2025 Report (Payment Year 2023).
- Applying varying CIF adjustments by long-term institutional (LTI) status and dual-eligible category (no Medicaid benefits, partial Medicaid benefits, full Medicaid benefits), based on MedPAC. *A Data Book: Health Care Spending and the Medicare Program*. July 2025. https://www.medpac.gov/wp-content/uploads/2025/07/July2025_ MedPAC_DataBook_SEC-2.pdf.
- Increasing the CIF adjustment applied with increasing length of MA enrollment. The CIF applied varied by MA tenure, based on Jacobs PD, Layton TJ. Identifying Coding Intensity in Medicare Advantage Through Switchers. *Health Serv Res*. Oct 2025;60(5):e14628. doi:10.1111/1475-6773.14628, as shown below:

| MA enrollment duration | Effective CIF | Jacobs-Layton Durational CIF Factor |
| --- | --- | --- |
| 1 year | 0.0% | 1.0000 |
| 2 years | 11.0% | 1.1240 |
| 3 years | 14.7% | 1.1720 |
| 4 years | 16.5% | 1.1975 |
| 5-6 years | 18.2% | 1.2230 |
| 7 years | 19.4% | 1.2413 |
| 8 years | 20.6% | 1.2597 |
| ≥9 years | 21.8% | 1.2780 |

We examined these coding scenarios individually. We found that our estimate of total favorable selection was somewhat robust to most coding scenarios. However, when we considered a higher total CIF (such as 10% or 15%) with zero coding intensity applied to first-year TM-to-MA switchers, we observed significantly lower estimates of total MA plan favorable selection. Favorable selection also declined by 2.6 pp when we assumed increasing coding intensity with MA tenure. Estimates increased by 1.3 pp under assumptions of higher coding intensity for dual eligibles. All favorable selection estimates were lower than our attempted replication of MedPAC’s 2025 estimate for payment year 2023 (8.6%).

***Sensitivity of Estimated Total Favorable Selection by Coding Intensity Scenario, 2020-2022***

| Coding Scenario | Effective CIF^*^ | Estimated Impact of Favorable Selection on MA Payments (%) | Percentage Point (pp) Difference Compared to This Study’s Estimate |
| --- | --- | --- | --- |
| CIF Levels Applied Uniformly to Switchers and Non-Switchers | 5.9%, 10.0%, 14.7% ^α^, 15.0% | 4.9% to 5.8% | 0.0 pp |
| CIF (5.9%) Applies to CMS-HCC Only; Not to First-Year MA^§¢^ | 5.9% | 4.5% to 5.5% | -0.4 pp |
| CIF (10.0%) Applies to HCC Only; Not to First-Year MA^§¢^ | 10.0% | 4.1% to 5.2% | -0.6 pp |
| CIF (15.0%) Applies to HCC Only; Not to First-Year MA^§¢^ | 15.0% | 3.6% to 4.8% | -1.0 pp |
| Higher Coding for Dual Eligibles^#^ (12.7% for LTI, 15.5% for non-duals, 30.1% for partial duals, 20.8% for full duals) | 17.7% to 17.9% | 5.7% to 6.6% | +0.8 pp |
| Increasing with enrollment^&¢^ | 18.0% | 2.0% to 3.2% | -2.6 pp |

^*^ Indicates the average CIF applied over the full MA population.

^§^ CIF not applied to demographic component of risk score.

^¢^ CIF not applied to members in their first year after switching from TM.

^α^ MedPAC calculated the impact of the coding adjustment as the MA coding intensity estimate relative to TM, multiplied by the coding adjustment (1.16). Thus, we divided MedPAC’s estimate of 17.0% by the 1.16 coding adjustment to align the interpretation across our analyses.

^#^ Calculation for coding intensity among dual eligibles based on MedPAC. *A Data Book: Health Care Spending and the Medicare Program*. July 2025. https://www.medpac.gov/wp-content/uploads/2025/07/July2025_ MedPAC_DataBook_SEC-2.pdf, Chart 9-9. We assigned 12.7% coding intensity for long-term institutionalized beneficiaries (regardless of dual status), 15.5% to enrollees with no Medicaid benefits, 30.1% to those with partial Medicaid benefits, and 20.8% to those with full Medicaid benefits.

^&^ CIF applied varied by MA tenure, based on Jacobs PD, Layton TJ. Identifying Coding Intensity in Medicare Advantage Through Switchers. *Health Serv Res*. Oct 2025;60(5):e14628. doi:10.1111/1475-6773.14628.

Together, these analyses probe the implicit assumption that remaining encounter‑data biases affect switchers and non‑switchers similarly. However, we note that there is variability and uncertainty in how the estimated degree of MA coding intensity can be applied at the beneficiary level; our analysis examines a limited set of possible scenarios.

*Plan and Geographic Mix Adjustment*

Our calculation of favorable selection includes standardizing spending for differences in plan mix (i.e., the share of beneficiaries enrolled in Health Maintenance Organization (HMO), Preferred Provider Organization (PPO), and other product lines) by reweighting non‑switchers to match the switcher distribution. We also standardized county‑level costs using 2022 CMS Ratebook area adjustment factors.

Our objective is to assess favorable selection by identifying where the risk adjustment model may incompletely capture beneficiary costs at the point of MA enrollment. Thus, our post-hoc standardizations for plan mix and geography aim to account for factors that the risk model is not designed to capture and that may influence observed risk‑adjusted spending independently of selection. These factors likely reflect post‑enrollment features of MA plans – such as utilization management, network design, and benefit structure – that can systematically affect utilization and spending. Differences in plan mix and geography between switchers and non‑switchers may therefore generate differences in observed risk‑adjusted costs that are not attributable to favorable selection but rather to the distribution of MA plan types and counties in which beneficiaries are enrolled.

Plan mix and geographic adjustments serve as a post‑hoc standardization to isolate selection effects from post‑enrollment plan influences that could otherwise bias inference. Importantly, this adjustment does not redefine the estimate but instead aims to ensure that differences in observed risk‑adjusted spending across groups are not driven by compositional differences in MA plan enrollment or county-level variation in MA enrollment. While favorable selection cannot be fully separated from plan‑driven influences on utilization, risk scores, and risk‑standardized spending, these adjustments are designed to reasonably address these factors.

To evaluate how plan mix and geographic adjustments influence our estimates, we rerun the analyses without these adjustments and compare the resulting estimates of favorable selection on MA payments.

Our results suggest that without plan mix and geographic adjustments, the estimated impact of favorable selection of MA payments increases by 2.6 to 3.4 percentage points (from 4.9%-5.8% to 6.6%-8.8%).

***Impact of Favorable Selection on MA Payments Without Plan Mix or Geographic Adjustment^*^***

***
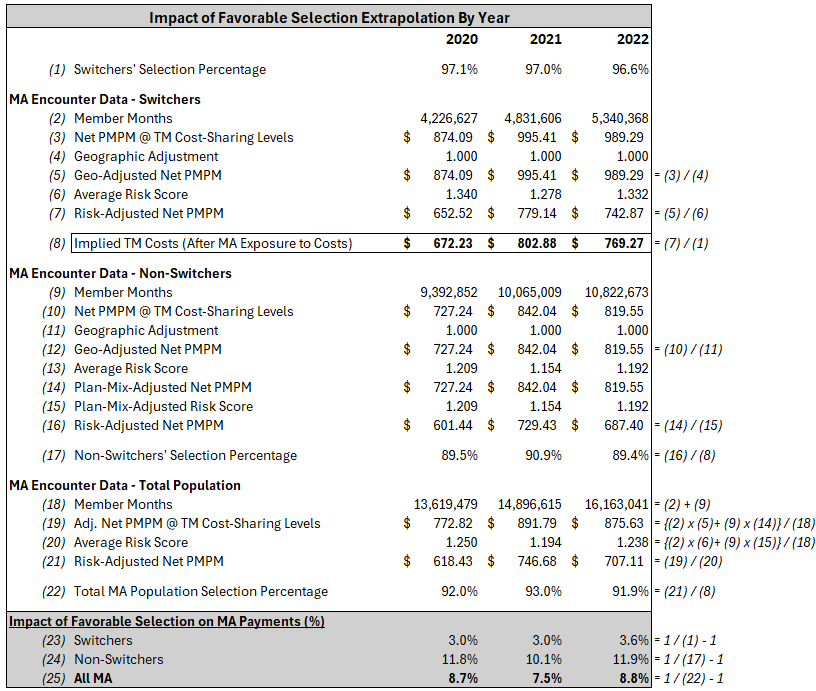
***

^*^ All MA risk scores expressed prior to CMS 5.9% Coding Intensity Factor adjustment.

***Impact of Favorable Selection on MA Payments With Plan Mix Adjustment Only (No Geographic Adjustment)^*^***

***
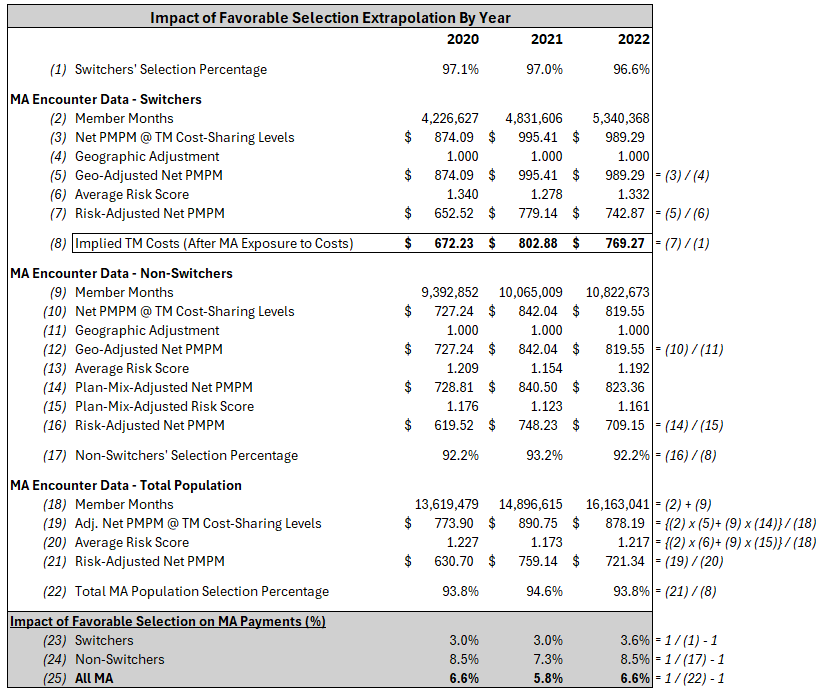
***

^*^ All MA risk scores expressed prior to CMS 5.9% Coding Intensity Factor adjustment.

***Impact of Favorable Selection on MA Payments With Geographic Adjustment Only (No Plan Mix Adjustment)^*^***

***
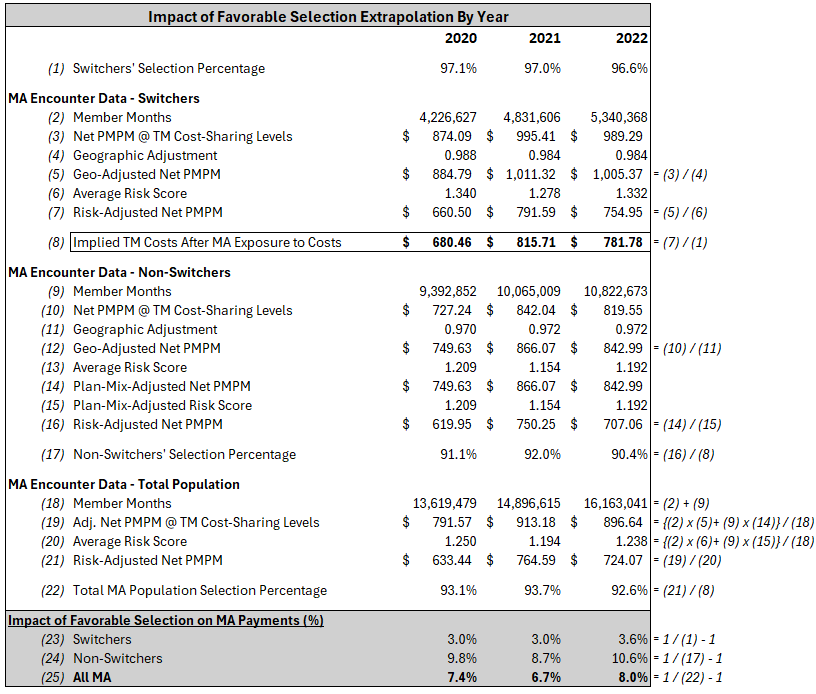
***

^*^ All MA risk scores expressed prior to CMS 5.9% Coding Intensity Factor adjustment.

**Section 5: Exploratory Post-Hoc Analyses**

*Impact of Controlling for Age Using MedPAC’s Extrapolation Approach*

MedPAC’s 2025 method for extrapolation assumed that switcher and non-switcher populations had the same selection patterns for beneficiaries in the same year of MA entrance and decedent status. However, our analysis of TM claims and risk score showed that risk-adjusted cost varied within decedent groups, with noticeably higher risk-adjusted costs observed for younger beneficiaries with the same decedent status. This finding suggested MedPAC’s extrapolation methods could improve its accuracy if matching on beneficiary age, in addition to the variables already being considered. To better understand the effect of accounting for more granular population differences, we attempted to replicate MedPAC’s extrapolation approach with one key difference – in addition to matching on MA entry year and decedent status, we also controlled for age. In addition, this approach enables us to quantify the magnitude of this effect using a methodology that is unaffected by utilization management and coding intensity.

Our attempted replication of MedPAC’s 2025 extrapolation methodology, incorporating age bands, in addition to MA entry year and decedent status yielded a total MA favorable selection estimate in 2022 of 5.6%, compared to 8.6% without the inclusion of age bands. This suggests that controlling for differences in risk-adjusted spending by age within decedent groups has a substantial impact on attenuating estimates of favorable selection.

| **Estimated Impact of Favorable Selection on MA Payments (%)** | **Percentage Point Difference Compared Our Attempted Replication of MedPAC’s 2025 Estimate (Payment Year 2022)^*^** | **Percentage Point Difference Compared to This Study’s Estimate^#^** |
| --- | --- | --- |
| 5.6% | -3.0 pp | -0.2 pp |

^*^ We compare to our attempted replication of MedPAC’s estimate in 2022 (8.6%), not MedPAC’s published amounts.

^#^ We compare to this study’s estimated impact of favorable selection on MA payments prior to applying a plan-mix adjustment in 2022 (6.2%).

1. Prior to applying CMS’s 5.9% CIF, the observed MA risk score exposure is 1.147. To apply the CIF, we scale this value by 94.1% (i.e., the complement of 5.9%, obtained by computing 100% - 5.9%), which yields an adjusted exposure of 1.079 (1.147 x 0.941). [↑](#footnote-ref-1)
2. [Announcement of Calendar Year (CY) 2022 Medicare Advantage (MA) Capitation Rates and Part C and Part D Payment Policies](https://www.cms.gov/files/document/2022-announcement.pdf), p60 - CMS applied a uniform 5.9% coding adjustment to MA plan risk scores to account for coding differences in 2022. [↑](#footnote-ref-2)
3. [MedPAC March 2023 Report to the Congress: Medicare Payment Policy](https://www.medpac.gov/wp-content/uploads/2023/03/Ch11_Mar23_MedPAC_Report_To_Congress_SEC.pdf), p 352 [ref figure 11-6]. [↑](#footnote-ref-3)
